# Uncovering physical activity trade-offs in transportation policy: A spatial agent-based model of Bogotá, Colombia

**DOI:** 10.1186/s12966-024-01570-1

**Published:** 2024-05-08

**Authors:** Ivana Stankov, Jose D. Meisel, Olga Lucia Sarmiento, Xavier Delclòs-Alió, Dario Hidalgo, Luis A. Guzman, Daniel A. Rodriguez, Ross A. Hammond, Ana V. Diez Roux

**Affiliations:** 1 Urban Health Collaborative, Dornsife School of Public Health, Drexel University, 3600 Market St, 7th Floor, Philadelphia, PA 19104 USA; 2 UniSA Allied Health & Human Performance, University of South Australia, North Terrace, Adelaide, SA 5000 Australia; 3 Facultad de Ingeniería, Universidad de Ibagué, Carrera 22 Calle 67, 730001 Ibagué, Colombia; 4 Social and Health Complexity Center, Universidad de Los Andes, Bogotá, Colombia; 5 Department of Public Health, School of Medicine, Universidad de Los Andes, Bogotá, Colombia; 6 Research Group On Territorial Analysis and Tourism Studies (GRATET), Department of Geography, Facultat de Turisme I Geografia, Universitat Rovira I Virgili, C/ Joanot Martorell, 15, 43480 Vila-Seca, Spain; 7 Department of Industrial Engineering, Pontificia Universidad Javeriana, Bogotá, Colombia; 8 Grupo de Sostenibilidad Urbana y Regional, SUR. Department of Civil and Environmental Engineering, Universidad de los Andes, Bogotá, Colombia; 9 Department of City and Regional Planning and Institute of Transportation Studies, University of California, Berkeley, 228 Bauer-Wurster Hall #1820, Berkeley, CA 94720-1820 USA; 10 The Brookings Institution, 1775 Massachusetts Avenue, N.W., Washington, DC USA; 11 Brown School at Washington University in St. Louis, 1 Brookings Dr, St. Louis, MO 63130 USA; 12 The Santa Fe Institute, 1399 Hyde Park Road, Santa Fe, NM 87501 USA

**Keywords:** Complex systems, Agent-based model, Transportation policy, Health inequities, Time scarcity, Physical activity

## Abstract

**Background:**

Transportation policies can impact health outcomes while simultaneously promoting social equity and environmental sustainability. We developed an agent-based model (ABM) to simulate the impacts of fare subsidies and congestion taxes on commuter decision-making and travel patterns. We report effects on mode share, travel time and transport-related physical activity (PA), including the variability of effects by socioeconomic strata (SES), and the trade-offs that may need to be considered in the implementation of these policies in a context with high levels of necessity-based physical activity.

**Methods:**

The ABM design was informed by local stakeholder engagement. The demographic and spatial characteristics of the in-silico city, and its residents, were informed by local surveys and empirical studies. We used ridership and travel time data from the 2019 Bogotá Household Travel Survey to calibrate and validate the model by SES. We then explored the impacts of fare subsidy and congestion tax policy scenarios.

**Results:**

Our model reproduced commuting patterns observed in Bogotá, including substantial necessity-based walking for transportation. At the city-level, congestion taxes fractionally reduced car use, including among mid-to-high SES groups but not among low SES commuters. Neither travel times nor physical activity levels were impacted at the city level or by SES. Comparatively, fare subsidies promoted city-level public transportation (PT) ridership, particularly under a ‘free-fare’ scenario, largely through reductions in walking trips. ‘Free fare’ policies also led to a large reduction in very long walking times and an overall reduction in the commuting-based attainment of physical activity guidelines. Differential effects were observed by SES, with free fares promoting PT ridership primarily among low-and-middle SES groups. These shifts to PT reduced median walking times among all SES groups, particularly low-SES groups. Moreover, the proportion of low-to-mid SES commuters meeting weekly physical activity recommendations decreased under the 'freefare' policy, with no change observed among high-SES groups.

**Conclusions:**

Transport policies can differentially impact SES-level disparities in necessity-based walking and travel times. Understanding these impacts is critical in shaping transportation policies that balance the dual aims of reducing SES-level disparities in travel time (and time poverty) and the promotion of choice-based physical activity.

**Supplementary Information:**

The online version contains supplementary material available at 10.1186/s12966-024-01570-1.

## Background

Urbanization has increased rapidly worldwide with transportation recognised as an important feature of urban life. Transportation has been linked to health – directly and indirectly – through numerous pathways. It catalyzes opportunities for generating income, a significant determinant of health and wellbeing, by facilitating access to employment and educational opportunities. Transportation also enables access to health-related resources such as health care services, as well as facilitating social interactions [1]. At the same time, motorized forms of transportation impact health through their generation of greenhouse gas emissions and air pollution, noise, and crash-related injuries [2]. Transportation choices can also impact health by enabling or hindering physical activity, affecting the capacity of populations to achieve recommended levels of activity that are supportive of health and wellbeing [3]. There is scope therefore for urban transportation policies to impact health outcomes while simultaneously promoting social equity and environmental sustainability [4].

A wide range of factors interact in complex ways to shape transportation mode choices including the activities that people engage in (work, school, shopping, recreation) and their relative location [5], social relationships and social norms [6] as well as perceptions of safety and experiences of crime [7–10]. Financial and time costs associated with a given form of transportation, including the ease of access to transportation infrastructure and services also affect travel choices [11–13]. Indeed, for some, transportation decisions are primarily motivated by economic factors [14]. For example, in lower-and-middle income countries (LMICs) such as those in Latin America and Africa, public transportation costs often exceed 25% of a low-income household’s total expenditure. In the face of such financial imposts, public transport is an untenable travel alternative for some [15], with the default ‘choice’ being to walk out of necessity. Such walking-for-transportation might be seen as advantageous for its health benefits, however, the ethics of promoting health behaviors that “result from nonautonomous, coercive circumstances (e.g., necessity-driven physical activity)” driven by spatial inequities in the location of jobs and affordable housing and limited access to affordable (or free) public transportation have increasingly been called into question (p.162 [14]) [16, 17]. There is therefore a need to better understand how transportation interventions can be leveraged to support physical activity but also address important inequities in travel times in LMICs.

In the rapidly growing cities of LMIC, particularly those in Latin America, transportation has emerged as a key policy priority because of its implications for physical activity promotion and environmental sustainability. Several types of policies have received special attention within the region, including, bus rapid transit (BRT) systems [18], Open Streets programs, which aim to promote active travel and recreation through temporary closures of roads to motorized modes of transportation [19], as well as the creation and expansion of cable cars that connect hillside neighbourhoods with central business districts [20, 21]. Many of these policies have sought to increase equity by targeting lower income areas most affected by lack of access to reliable and quality public transportation [22]. The success of these policies in addressing socio-spatial inequalities, however, has been mixed. This is because transportation policies operate in the context of urban systems which dynamically interact with and modify the effectiveness of these policies. Therefore, approaches that account for the complex interdependencies between urban factors and the functioning of these systems are needed to design interventions in ways that will maximise their intended effects.

Agent-based models (ABMs) are tools that can simulate the factors that shape transportation behavior. Specifically, they have the capacity to represent important attributes of the transportation environment and account for heterogeneous commuter characteristics and preferences that drive people’s decision-making in ways that are spatially explicit [23]. By characterizing the key variables, relationships, and feedback loops shaping transportation decisions, ABM provides an opportunity to explore the impact of transportation policies in a dynamic environment, on multiple outcomes and under varying conditions [24]. ABMs have increasingly been used to explore the potential impact of interventions promoting active travel, with a focus on health-behaviors such as physical activity and walking [13, 25, 26]. However, most of these applications have been abstract and not specifically developed for a LMIC urban setting [13].

We used an ABM to explore the impact of transportation interventions on mode share, travel times (comprising total and active time—which includes the walking segments of bus and BRT trips), and the World Health Organisation (WHO) [3] recommended levels of physical activity (at least 150 min per week) through transport in a prototypical Latin American city. Informed by input from stakeholders across three regions of Latin America, including policymakers, academic researchers, members of the private sector and civil society (e.g., nonprofits) who engaged in participatory group model building workshops [8], we developed an ABM of commuting behavior based on the city of Bogotá, Colombia, and used this model to simulate 1. fare subsidies designed to promote/increase public transportation, 2. congestion taxes that aim to limit/ disincentivize car use, and 3. combinations of fare subsidy and congestion tax policy scenarios. Given the high levels of inequality in Latin American cities, we report on the heterogeneity of policy effects across socioeconomic strata (SES).

## Methods

We describe key components of the model design using the ‘PARTE’ framework, which characterizes: Properties of agents; Actions or behaviors modelled; Rules that govern such behavior; Time; and the Environment in which agents are embedded [24]. Whenever possible, key components of the model were informed by the city of Bogotá, Colombia.

### Properties of agents and their actions

The ABM simulates the actions of 400,000 agents, which represent people, as they make decisions about how to commute to and from work each weekday. Each agent $i$ was assigned an SES, a gender, a home and workplace location based on their SES. Other attributes, including agents’ income ${w}_{i}$, car ${c}_{i}$, motorcycle ${m}_{i}$ and bike ownership ${b}_{i}$ were randomly assigned based on income distributions and vehicle ownership prevalence rates informed by their SES. Each agent was also assigned a set of friends or social contacts. These were modelled after a simplified small-world network which assumes that agents have friends (other agents in the model) that live (*n* = 3) and work (*n* = 3) close to the agent’s home and workplace (spatial clustering), as well as friends that live and work further away (*n* = 3) but are of the same gender (representing homophily). Agents were also assigned a safety risk sensitivity ${s}_{i}$, which characterizes an agent’s level of risk aversion (i.e., the higher the sensitivity, the higher an agent’s level of risk aversion), which varies by SES, and a memory of the crime/ victimization experiences ${SRlog}_{im}$ while travelling using each transportation mode $m$. These attributes, along with their friends’ experiences of crime/ victimization while commuting, determine an agents’ overall perception of the crime risk ${sr}_{im}$ associated with travelling via a particular mode of transportation $m$. Each agent also forms memories of the length of their commute ${Tlog}_{im}$ and the amount of physical activity ${PAlog}_{im}$ accrued on their journey. Table 1 describes agent attributes in greater detail.

**Table 1:** Summary of agent attributes at baseline, and the data/ empirical sources informing these

Parameter	Agent attribute definition and values	Data and empirical foundation
${SES}_{i}$	Socioeconomic strata (SES) Categorical ∈ [SES1, SES2, SES3, SES4, SES5, SES6]	Colombia’s national socioeconomic classification system is based on housing and neighbourhood-level characteristics. The strata range from lowest (SES1) to highest (SES6) and serve as a proxy for household income. Randomly assigned with proportion of residents belonging to each socioeconomic stratum (SES1 = 0.11; SES2 = 0.3; SES3 = 0.36; SES4 = 0.16; SES5 = 0.04; SES6 = 0.03) derived from the 2019 Bogotá Household Travel Survey [27]
${w}_{i}$	Daily personal income (COP/ day) $\left\{{w}_{i}\in {\mathbb{R}}\,|\ {w}_{i}>0\right\}$	Daily personal income, in Colombian Pesos per day. Separate personal income distributions were derived for each SES group using monthly household income and average household size data from the 2019 Bogotá Household Travel Survey [27]. Values were drawn at random from log normal (for SES 1 & 2) and normal distributions (for SES 3, 4 & 6) based on the agent’s SES. Values remain constant over time. For information on how these distributions were derived and their characterization, refer to Supplement S1a
${g}_{i}$	Gender Categorical $\in$ [male, female]	Randomly assigned with proportion of male (0.54) and female (0.46) residents derived from the 2019 Bogotá Household Travel Survey [27]
${c}_{i}$	Car ownership Categorical $\in$ [car, no car]	Randomly assigned with proportion of car, motorbike and bicycle owners in each SES group informed by 2019 Bogotá Household Travel Survey [27]. Values remain constant Proportion with **car**: SES1 = 0.13; SES2 = 0.21; SES3 = 0.39; SES4 = 0.64; SES5 = 0.81; SES6 = 0.80 Proportion with **motorbike**: SES1 = 0.17; SES2 = 0.17; SES3 = 0.13; SES4 = 0.06; SES5 = 0.06; SES6 = 0.05 Proportion with **bicycle**: SES1 = 0.24; SES2 = 0.35; SES3 = 0.41; SES4 = 0.43; SES5 = 0.45; SES6 = 0.41
${m}_{i}$	Motorbike ownership Categorical $\in$ [motorbike, no motorbike]	
${b}_{i}$	Bicycle ownership Categorical $\in$ [bicycle, no bicycle]	
${s}_{i}$	Safety risk sensitivity $$\begin{array}{l}\left\{{s}_{i}\in \left\{0.1, 0.2, 0.3\right\}|\ {SES}_{i}=1 \,or\ 2\right\}\\ \left\{{s}_{i}\in \left\{0.4, 0.5, 0.6, 0.7\right\}|\ {SES}_{i}=3 \,or\ 4\right\}\\ \left\{{s}_{i}\in \left\{0.8, 0.9, 1\right\}|\ {SES}_{i}=5 \,or\ 6\right\}\end{array}$$	Randomly drawn from uniform distribution of decimal values based on SES group. Represents level of risk aversion and remains constant over time. Assumed to be lowest among low SES agents due to lack of access to diverse forms of transportation and higher baseline exposure to crime which might result in relative desensitization to crime compared to higher SES groups
${SRlog}_{im}$	Memory of agent $i$’s experiences of crime/ victimization while travelling via mode $m$ Categorical $\in$ [0, 1]	Initialized as zero and updated each time agent $i$ travels using mode $m$. Characterizes agent $i$’s 120 most recent travel experiences using mode $m$ (as detailed in Supplement S1d)
${sr}_{im}$	Perceived risk to personal safety score of travel with mode $m$ $\left\{{sr}_{im}\in {\mathbb{R}}\,|\ 0\le {sr}_{im} \le 1\right\}$	Initialized as zero and updated each time agent $i$ travels using mode $m$, according to safety risk rule
$tval$	Value of time	Weight for the value of time in calibrated model = 3.5
$mval$	Value of money	Weight for the value of money set to 1
$exval$	Value of exercise	Weight for the value of exercise in calibrated model = 0.6
${Tlog}_{im}$	Memory of agent $i$’s travel time using mode $m$	Initialized as zero and updated each time agent $i$ travels using mode $m$. Characterizes agent $i$’s travel time over the course of the past 20 completed trips using mode $m$ (as detailed in Supplement S1d)
${PAlog}_{im}$	Memory of agent $i$’s physical activity using mode $m$	Initialized as zero and updated each time agent $i$ travels using mode $m$

### Actions

In the model, each time step is one day, representing the morning commute to work and afternoon commute back home (we assumed the same transportation mode in the afternoon as in the morning). We consider only single-mode trips involving travel by bus, BRT, car, motorcycle, bicycle, and walking. First, agents eliminate the modes which are unavailable to them (i.e., modes which they do not own—carpooling and rideshare are not considered in the model). Agents then consider the affordability of each available mode and exclude any modes deemed too expensive from their consideration (i.e., those modes that cost more than the agent’s daily income). Second, the level of personal safety associated with the remaining available modes is considered, and those considered to be too unsafe are excluded from the agent’s choice set with a certain probability. Agents evaluate personal safety by evaluating factors such as their own past experiences and the collective experiences of their friends using the safety risk rule (detailed below). Finally, the remaining modes are evaluated based on the out-of-pocket cost, travel time and exercise cost of travel via that mode. Agents evaluate all these factors using the mode choice rule (described below). The mode considered most favorable overall is ultimately selected for the entire journey (i.e., from home to work and back again) [11].

### Rules

#### Safety risk rule

We employed a probabilistic process to determine whether a given mode of transportation is considered too unsafe and therefore excluded as a potential travel option (Supplement S1b).

#### Mode choice rule

We used a travel demand discrete choice model to represent how agents decide among the modes available to them [11, 28]. The utility of each transportation mode is expressed as a monetary cost and considers the out-of-pocket, travel time and exercise cost of travel via that mode. Given that discrete choice models assume that people are rational decision-makers [11, 28], once agents calculate the utilities of all modes in their choice set, a deterministic cost-minimization process is used to determine the most attractive option and the mode that is ultimately chosen. That is, the mode with the lowest overall monetary cost (see Supplement S1c for further details). For simplicity, we assume that once an agent has decided how to travel to work on a given day, they use the same mode to return home at the end of the day (this includes the same bus and BRT stop). Moreover, we assume that agents will only consider the BRT stations closest to their home and workplace. Agents travelling by bus on the other hand, randomly select a bus stop within an 800 m radius of their home and workplace as points of ingress/ egress to the bus services [29]. This variability in the selection of bus stops is intended to account for day-to-day differences in the frequency and punctuality of bus services.

#### Model time & environment

**Fig. 1:**
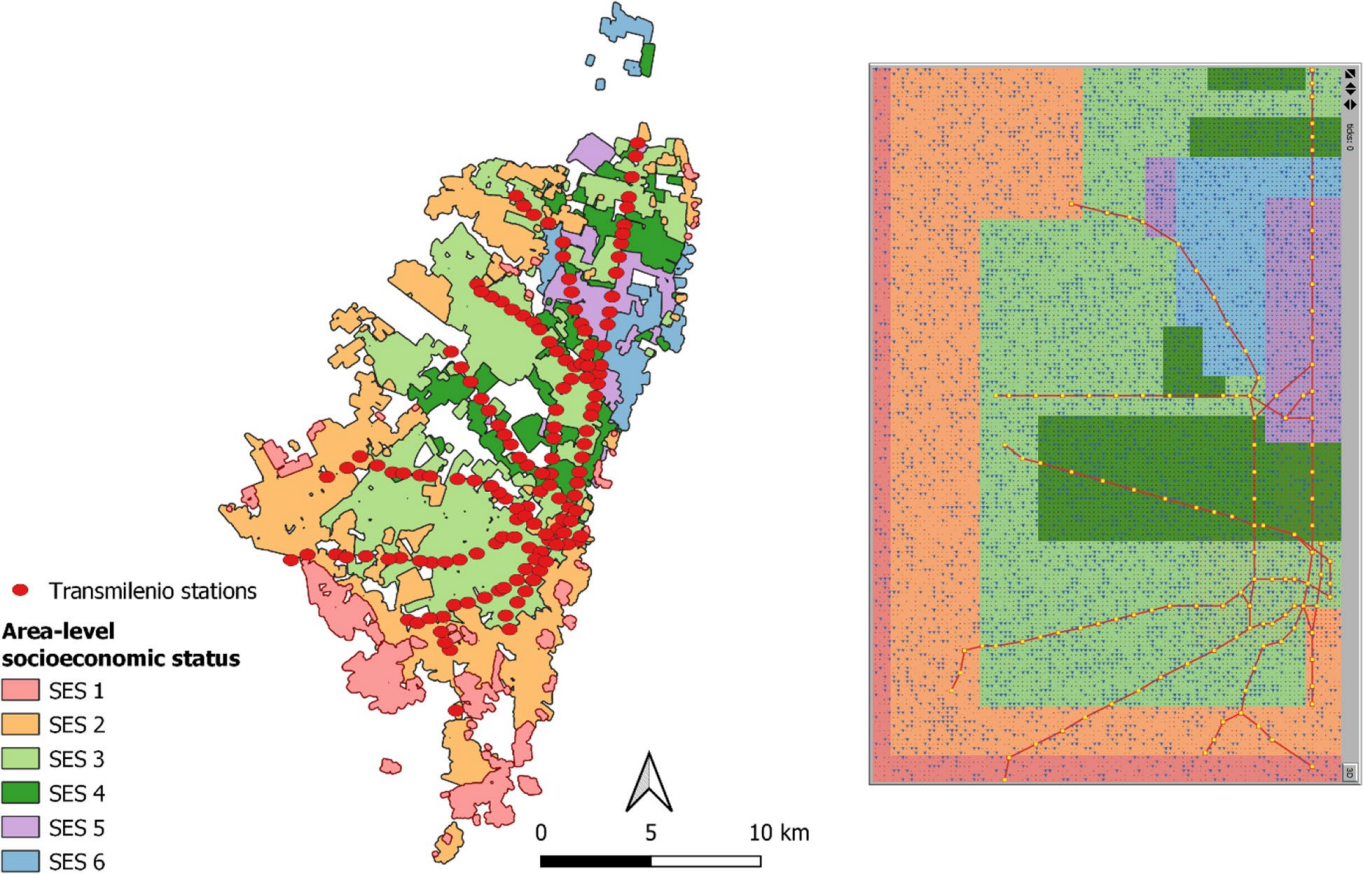
Map of the City of Bogotá showing the extent and distribution of the TransMilenio bus rapid transit system and the six SES strata in the city (left) along with the abstract representation of the city in the ABM environment (right)

In the model, each time step is one day which comprises the morning commute to work and the afternoon commute back home. Environmental factors (e.g., access to public transportation, travel time) play an important role in shaping commuter decision-making and behavior. The ABM environment was therefore designed to provide a stylistic representation of the City of Bogotá, including its public transportation system and distribution of workplaces and people resident in socioeconomically diverse areas of the city. The environment was set up as a 160 × 105 surface, where each unit represents a 100 × 100 m block, designed to represent the 168km^2^ area of the city. Geographic data [30] was used to inform an abstract representation of the spatial extent and distribution of the six SES strata and the BRT system in the city (Fig. 1). Agents were randomly assigned a home location, while ensuring that the population density within each SES zone aligned with the data [27]. The empirical literature was further used to inform the location of the central business district (CBD), along with three different work zones and the density of workplaces in each of these zones [31]. Agents were assigned to workplace locations based on their income level [32, 33]. Other environmental factors include waiting time distributions for bus and BRT users, mode travel speeds and costs and prevalence rates of personal crime associated with each mode of transportation. Agents’ social contacts were modelled using a small-world network which assumes that agents have friends (other agents in the model) that live (*n* = 3) or work (*n* = 3) close to the agent’s home and workplace, respectively, as well as friends that live and work further away (*n* = 3 agents of the same gender selected at random). These factors are described in Table 2 and elaborated further in Supplement 2.

**Table 2:** Summary of environmental attributes and the data/ empirical sources informing these

Parameter	Environment attribute definition and values	Data and empirical foundation
${sl}_{m}$	Slope penalty $\left\{{sl}_{m}\in {\mathbb{R}}\,|\ {sl}_{m}>0\right\}$	The slope penalty was applied to agents traveling from peripheral areas of the city to/ from the central business district (CBD) since these trips involve at least one journey negotiating an uphill slope (0.5%). The use of this penalty aligns with the empirical literature which found that the attractiveness of both walking and bicycling decreases with increasing slope [34, 35]. Remains constant For walking and bicycle trips from peripheral areas of the city to/from CBD slope penalty ${sl}_{w}=1.05$; ${sl}_{b}=1.125$. For all other walking and bicycle trips, no penalty is applied i.e., ${sl}_{m}=1$. See Supplement S2d for more information
${wt}_{im}$	Waiting time distributions, for bus and BRT, by SES $\left\{{wt}_{im}\in {\mathbb{R}}\,|\ {wt}_{im}>0\right\}$	Bus and BRT waiting times (in minutes) by SES, assumed to be normally distributed with mean ($\widetilde{x}$), sd, maximum and minimum (set to zero) informed by data derived from the 2019 Bogotá Household Travel Survey [27]. Agents draw a new value each time step **Bus:** SES1 [$\widetilde{x}$ =18.6, sd = 13.6, max = 90]; SES2 [$\widetilde{x}$ =15.1, sd = 11.9, max = 127]; SES3 [$\widetilde{x}$ =14.8, sd = 12.1, max = 120]; SES4 [$\widetilde{x}$ =12.0, sd = 8.8, max = 60]; SES5 [$\widetilde{x}$ =11.6, sd = 9.7, max = 40]; SES6 [$\widetilde{x}$ =11.9, sd = 8.4, max = 40]
		**BRT:** SES1 [$\widetilde{x}$ =16.5, sd = 10.9, max = 60]; SES2 [$\widetilde{x}$ =14.5, sd = 10.6, max = 75]; SES3 [$\widetilde{x}$ =12.5, sd = 9.3, max = 90]; SES4 [$\widetilde{x}$ =11.0, sd = 7.5, max = 45]; SES5 [$\widetilde{x}$ =9.9, sd = 5.9, max = 30]; SES6 [$\widetilde{x}$=8.6, sd = 5.4, max = 20] All waiting times are expressed in minutes
${s}_{im}$	Mode speeds $\left\{{s}_{im}\in {\mathbb{R}}\,|\ {s}_{im}>0\right\}$	Average car [36], bus [37], BRT [38], bike [39] and walking [40, 41] speeds informed by the literature and converted to meters per minute in the model. Given the absence of information concerning motorcycle speeds in Bogotá, we assumed motorcycles travel slightly faster than cars given their ability to weave between cars **Car** = 35 km/h (583 m/min); **Motorcycle** = 40 km/h (667 m/min); **Bus** = 13.7 km/h (228 m/min); **BRT** = 26 km/h (433 m/min); **walking** = 4.8 km/h (80 m/min); **bicycle** = 17 km/h (283 m/min)
${C}_{m}$	Mode cost $\left\{{C}_{m}\in {\mathbb{R}}\,|\ {C}_{m}>0\right\}$	Mode costs include fuel costs [42] (for car and motorbike, which are calculated based on the total travel distance), fare costs for bus and BRT (full price and subsidized fares for those meeting criteria for SISBEN (Sistema de Selección de Beneficarios) subsidy ~ 10.2% of population, excluding older people and people with disabilities (assumed to be poorest 10.2% of agents)) [43, 44], and parking costs [45] (for bike, car and motorbike, which depend on level of parking demand in agent’s work zone, and level of parking service—parking at a facility, on a concrete floor or on a grass floor—which is randomly chosen each day) Fuel costs: **motorcycle** & **car** = 0.23858 COP/m Fare costs: a) Full price fares—**BRT** = 2400COP; **bus** = 2200COP/ trip; b) SISBEN subsidized fares—**BRT** = 1991COP; **bus** = 1825/trip (average per cost estimated by assuming 260 business days/year ~ 22 days/month or 44 return trips, up to 30 trips at SISBEN subsidized price (BRT = 1800COP; bus = 1650COP [43]) and remaining trips at full price) Parking costs: **bike** = 4800COP/day; **motorcycle**: high demand $\in [17760 \,24960 \,35520]$ & low demand $\in [14400 \,19680 \,28320]$ facilities (COP/day) **Car**: high demand $\in [25440 \,35520 \,50400]$ & low demand $\in [20160 \,28320 \,40320]$ facilities (COP/day)
$Pmpp$	Percentage of motorcyclists paying for parking $\left\{Pppm\in {\mathbb{R}}\,|\ Pppm>0\right\}$	The percentage of motorcyclists paying for parking in the model is 55%, as determined though model calibration. We assumed that only a fraction of motorcyclists use paid parking facilities given that illegal parking, including frontage parking – parking on sidewalks and spaces between the buildings and the street – is not uncommon
${RC}_{mg}$	Rate of personal crime on mode $m$ for agent with gender $g$ $\left\{{RC}_{mg}\in {\mathbb{R}}\,|\ {0<RC}_{mg}<1\right\}$	The prevalence of personal crime was informed by 2015 crime [46–50] and trip data [51]. Crime data were not available for bicycle, car and motorcycle trips so we made assumptions about the crime prevalence relative to the other modes. Furthermore, we apportioned these overall crime prevalence rates to men and women, based on data from mobility surveys [51]. (See Supplement S2e for more information). Crime prevalence rates by mode remain constant **Car**: male = 0.007% & female = 0.008%; **motorcycle**: male = 0.007% & female = 0.003%; **BRT**: male = 0.027% & female = 0.048%; **Bus**: male = 0.011% & female = 0.017%; **bike**: male = 0.370% & female = 0.130%; **walking**: male = 0.328% & female = 0.590%
${n}_{mz}$	Number of bus and BRT stops, by SES stratum $z$ $\left\{{n}_{mz}\in {\mathbb{R}}\,|\ {n}_{mz}>0\right\}$	Geospatial data were used to derive the number of bus [52] and BRT [53] stops in each SES stratum (see Supplement S2e for more information). For simplicity, bus stops were randomly distributed within each SES stratum, while BRT stops were roughly distributed according to BRT route maps **Bus** stop count: SES1 = 620; SES2 = 2,323; SES3 = 2,835; SES4 = 769; SES5 = 327; SES6 = 236; Total = 7,110 **BRT** stop count: SES1 = 0; SES2 = 12; SES3 = 76; SES4 = 27; SES5 = 8; SES6 = 3; Total = 126
${n}_{wz}$	Number of workplaces $w$, by SES stratum $z$ and within the CBD $\left\{{n}_{wz}\in {\mathbb{R}}\,|\ {n}_{wz}>0\right\}$	Estimated using data from the empirical literature [31]. For simplicity workplaces were randomly distributed within each SES stratum and the central business district (see Supplement S2c for more information) Workplace count: CBD = 16,495; SES1 & 2 = 3,705; SES3 = 11,351; SES4, 5 & 6 = 15,188

### Model calibration

The relative value of time, money, and exercise in the decision-making process could not be informed by empirical data. As such, plausible values of these parameters were determined through calibration. Given the limited flexibility of Netlogo and available memory, we were unable to apply pre-established optimization algorithms. As such, we manually co-varied each unknown parameter over a defined range to identify a set of values that maximized the alignment of model’s simulated outputs with city-level mode share patterns observed in the 2019 Bogotá Household Travel Survey. A secondary aim was to align the relative prevalence of transportation mode use by SES. Where multiple plausible configurations of values were identified, we assigned higher values to time and money relative to exercise, as these former factors are normatively featured in models for transportation decision-making [11].

Over several calibration attempts, the model consistently simulated levels of bicycle and BRT use that were significantly higher than observed in the Bogotá Household Travel Survey. As such, we introduced two additional parameter weights to represent factors that could variously impact decision-making related to these modes (e.g., weather, road traffic safety, shower facilities, crowding), but which were not accounted for in our utility functions due to data limitations. These parameter weights were calibrated in addition to the weights characterizing the relative influence of time, money, and exercise.

### Sensitivity analysis

A sensitivity analysis was conducted to test the model’s sensitivity to uncertainty in the specification of attributes relating to the ‘safety risk rule’, which underpins agent’s considerations of personal safety from crime. Specifically, local sensitivity analysis was performed on the crime prevalence and safety risk sensitivity distribution as described in Supplement 3.

### Simulated policies

We simulated two different types of policies, including two levels of a fare policy impacting the public transportation system (i.e., buses and BRT), and two levels of a congestion tax seeking to reduce car use and thereby congestion. These policies were selected based on their prioritization by policymakers in the region [8] and discussions with the research team which comprised transportation experts with an intimate knowledge of the policy landscape through their work with transportation policymakers in Bogotá. We also simulated the impact of the combined implementation of all possible fare and congestion tax scenarios on mode share, as well as active and total travel time, overall and by SES. For fare policies, we modelled a 30 percent reduction in the fare price as well as an ambitious fare scenario where public transportation (i.e., all buses and BRT) were made free for all residents. For congestion taxes, we modelled two levels benchmarked to congestion taxes implemented elsewhere. While there exists significant variation in how these taxes have been implemented across cities, they tend to fall into two general types: 1) by delineating an area or set of roads, where drivers are charged as they move through the area based on distance driven (e.g., Singapore) or 2) by delineating a ‘cordon area’ or ‘congestion charge zone’ and charging drivers a daily fee for entering into that area (e.g., London and Stockholm). Across these cities, the magnitude of the tax has ranged anywhere between 5% and 8.4% of residents’ average income for travel during peak hours. We modelled a congestion tax in the form of a daily fee charged to car users driving anywhere in the city. We used the midpoint of the proportions charged in other cities as a benchmark to inform the fee charged in one of our simulated scenarios. That is, we calculated 6.5% of the average income in Bogotá, which was around 2,000 COP, and used this value in one of the simulated congestion tax scenarios. We also explored a more extreme tax scenario where drivers pay 20,000 COP (a fee that is 10 times higher than the benchmark) each day they choose to drive.

## Results

**Fig. 2:**
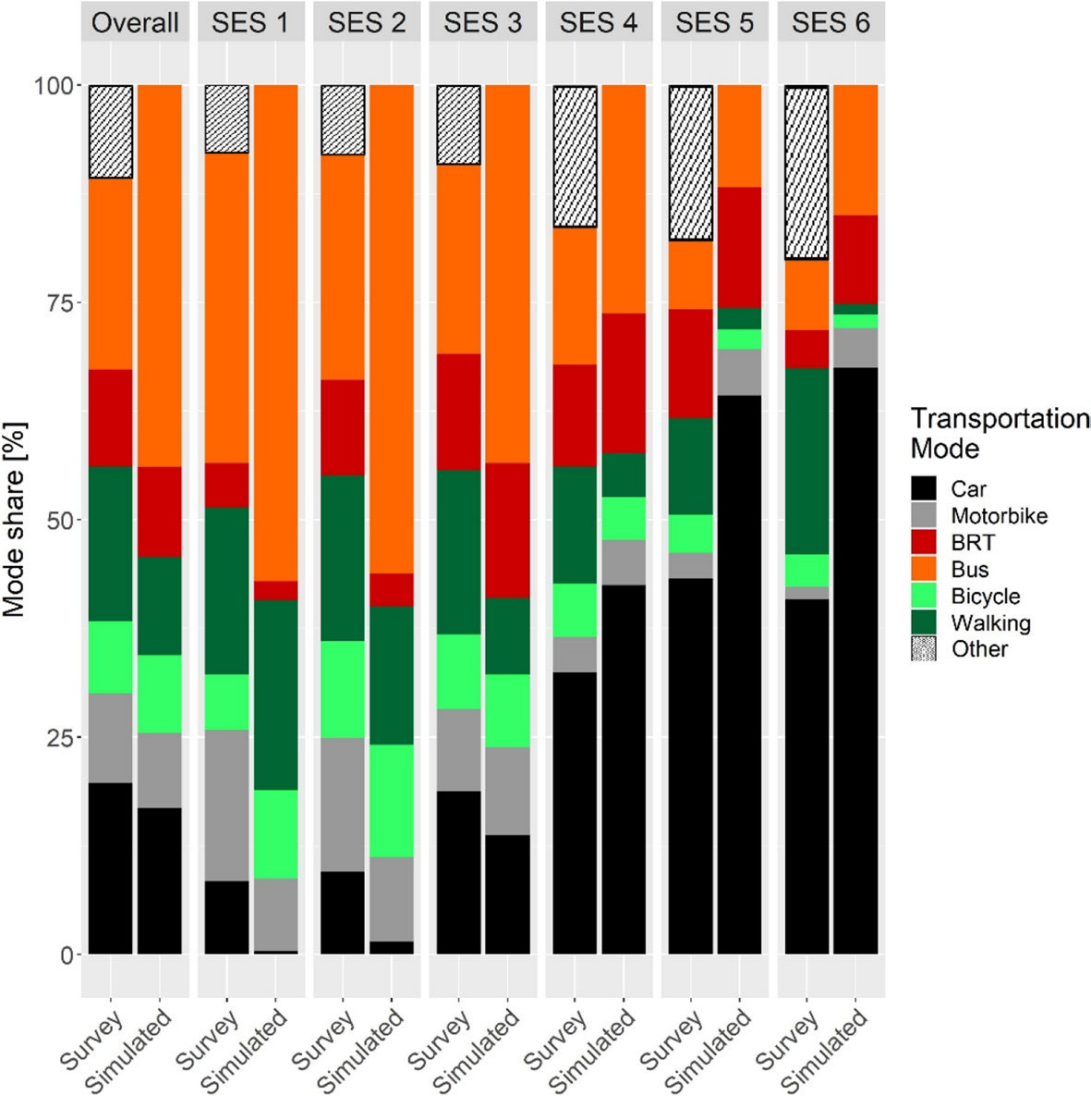
Baseline calibrated model fit to percent mode share data from the 2019 Bogotá Household Travel Survey. Each facet represents a population subgroup, ranging from overall, city-level mode share (facet 1) to SES-level mode share (columns 2–7). Within each facet, the left-hand bar depicts mode share patterns observed in the 2019 Bogotá Household Travel Survey, while the right-hand bar shows the model simulated mode share. The different colors represent different modes of transport. Notably, the Travel Survey also captures ‘Other’ modes of travel used in Bogotá (e.g., cable car, taxi etc.) that were not simulated by the model

The calibrated model demonstrated a good fit to population-level patterns of mode share in Bogotá, and a fair fit to mode share patterns by SES (Fig. 2). Notably, among SES 1–3 (lower SES), the model tended to overestimate the prevalence of bus use and underestimated the proportion of car drivers. Conversely, among SES 4–6 (higher SES), we observed an overestimation of car driving and an under-representation of walking only trips.

The findings of the sensitivity analysis suggest that model outcomes are robust and insensitive to uncertainty in the crime prevalence estimates, and the specification and assignment of risk sensitivities by SES (Supplement 3).

### Mode share

#### Fare subsidies

**Fig. 3:**
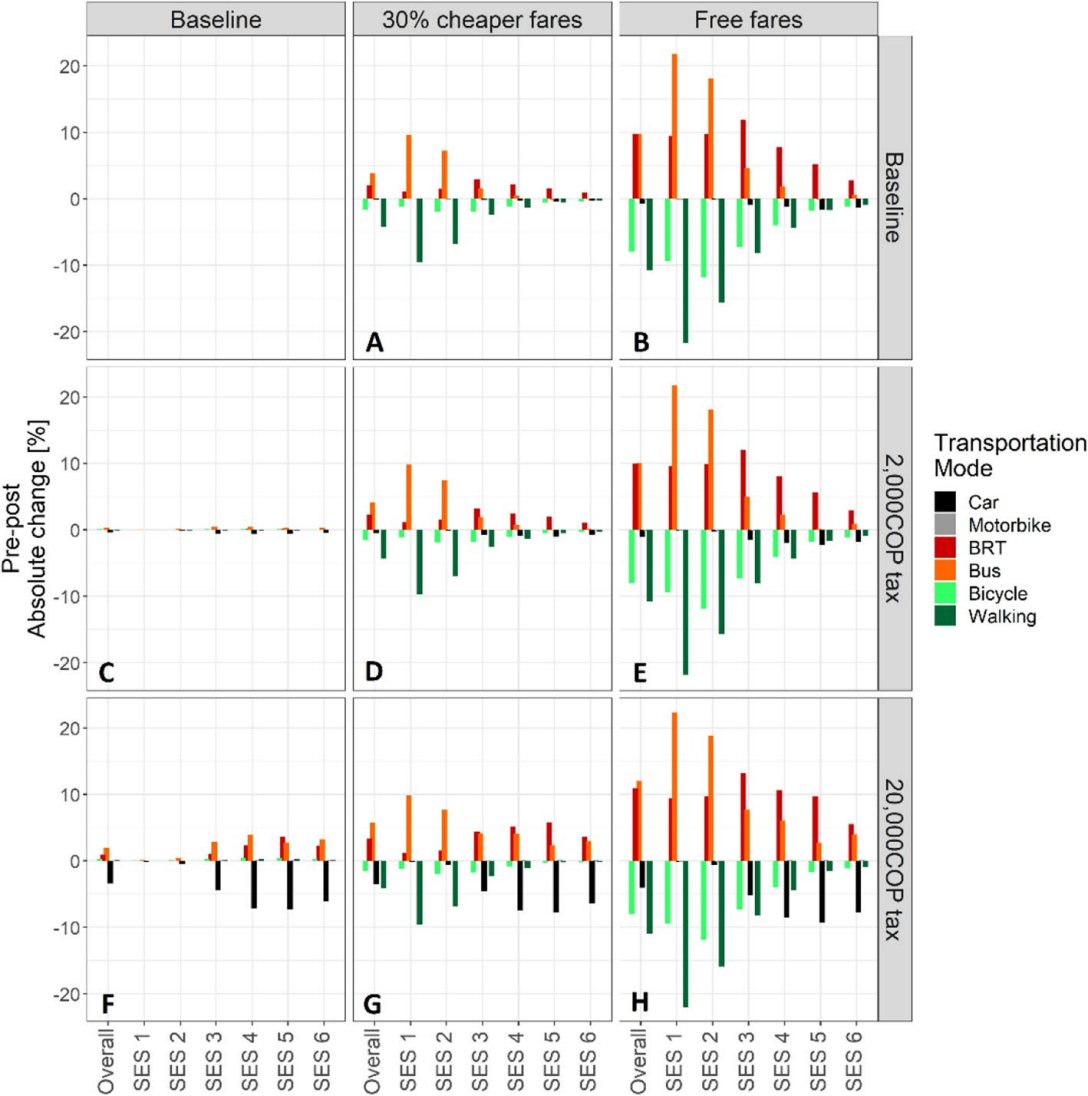
Absolute percent change in mode share (y-axis), by mode, overall and by SES (x-axis) following the implementation of **A** a 30% fare subsidy only; **B** free bus and BRT travel for all, a congestion tax of 2,000 COP only (**C**) and 20,000 COP only (**F**), and a combination of these fare subsidies and congestion taxes (**D**, **E**, **G**, **H**)

The free fare policy was approximately twice as effective as the 30% fare subsidy in motivating changes in mode share (Fig. 3A & B). Both fare subsidies promoted bus and BRT use by incentivizing bicycle users and those walking-to-work to shift to public transportation. A socioeconomic gradient in the effects of the fare subsidies was observed, with agents from the lowest SES strata (SES1 and 2) experiencing the largest shifts from walking to bus use while agents from higher SES-categories (SES 3 - 6) predominantly shifted from walking and bicycling to BRT use. The fare policies alone did not significantly impact car use.

#### Congestion taxes

The congestion taxes were generally less effective in motivating changes in mode share compared to the fare policies (Fig. 3C & F). The low-level congestion tax (2,000 COP) had no impact on mode share, while the high-level congestion tax (20,000 COP) was approximately as effective as the 30% fare subsidy in shifting mode share. Specifically, the high congestion tax promoted shifts from car to bus and BRT use, predominantly among agents in SES 3 - 6.

#### Combination policies

An additive effect was observed in scenarios combining the implementation of fare subsidies and congestion taxes. Combining the low-level congestion tax (2,000 COP) with the fare policies (Fig. 3D & E) did not promote mode share change above the levels observed with each fare policy alone. The high-level congestion tax (20,000 COP) combined with the fare policies (Fig. 3G-H), however, resulted in greater increases in bus and BRT use (than the fare policies alone) and a slightly greater reduction in car use (than the congestion tax alone).

### Active and total travel time

#### Fare subsidies

**Fig. 4:**
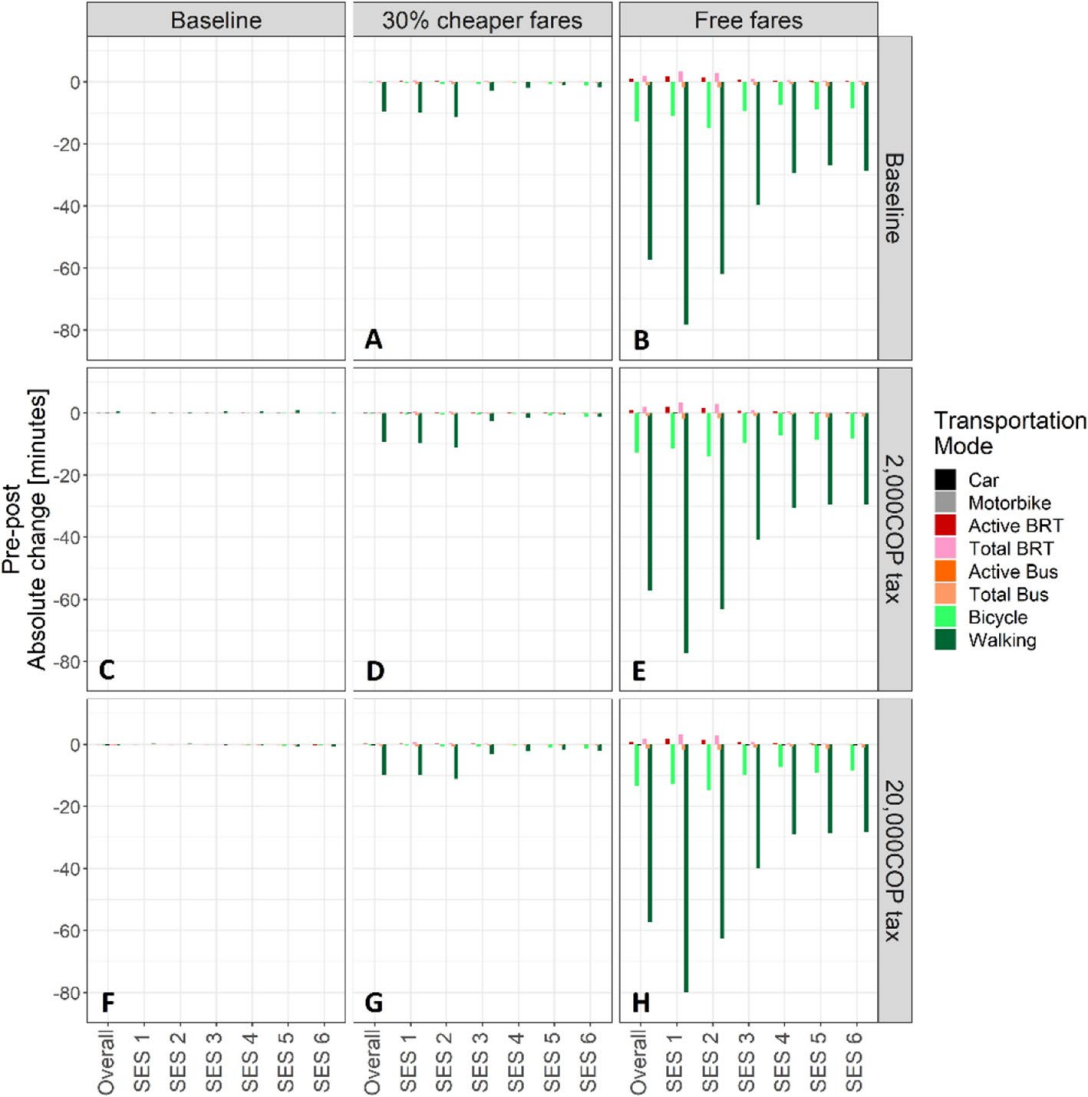
Absolute change in median active and total travel time, in minutes (y-axis), by mode, overall and by SES (x-axis) following the implementation of **A** a 30% fare subsidy only; **B** free bus and BRT travel for all, a congestion tax of 2,000 COP only (**C**) and 20,000 COP only (**F**), and a combination of these fare subsidies and congestion taxes (**D**, **E**, **G**, **H**)

The free fare policy resulted in a much greater reduction in median travel time compared to the 30% fare subsidy, particularly for walking and bicycle trips (Fig. 4A & B). A socioeconomic gradient in the effects of the fare subsidies was observed at both levels of the fare subsidy. The 30% fare subsidy did not impact median bicycle travel time but instead resulted in modest reductions in median walking time, overall and for SES groups 1–2. On the other hand, for the free fare subsidy, modest reductions in median bicycle travel time and sizable decreases in median walking time overall and across all SES groups were observed, particularly in SES 1-2. Notably, no significant changes in median total and active travel time were observed for the other modes.

**Fig. 5:**
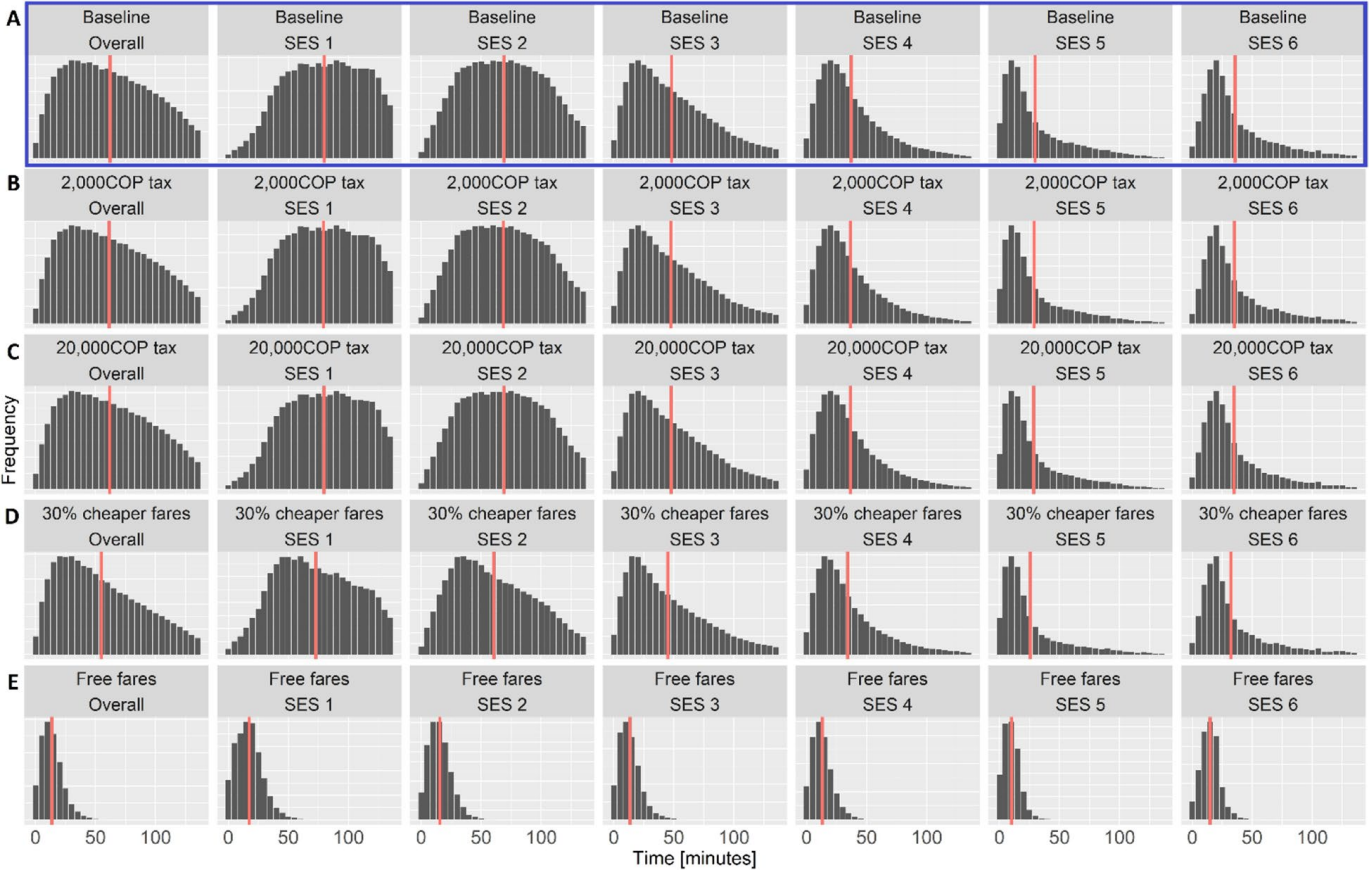
Distribution of times spent walking to work each day (red line signifies median), overall and by SES for each scenario; the baseline model (blue section **A** no policy intervention), the congestion taxes, including 2,000 COP (**B**) and 20,000 COP tax (**C**), and the fare subsidies; 30% fare subsidy (**D**); and free public transport for all users (**E**)

Both fare policies shifted the overall population-level distribution of walking to the left (Fig. 5D & E), by reducing the number of very long walking trips (≥ 60 min one-way). This reduction in walking was particularly pronounced among the lowest SES groups (SES 1–2).

#### Congestion taxes

The congestion taxes had no impact on median total and active travel time by mode and across SES (Fig. 4C & F).

#### Combination policies

The combined policies did not reduce median travel time beyond the levels observed with each fare policy alone (Figs. 3D-E & 4G-H).

### Weekly recommended physical activity guidelines

#### Fare subsidies

**Fig. 6:**
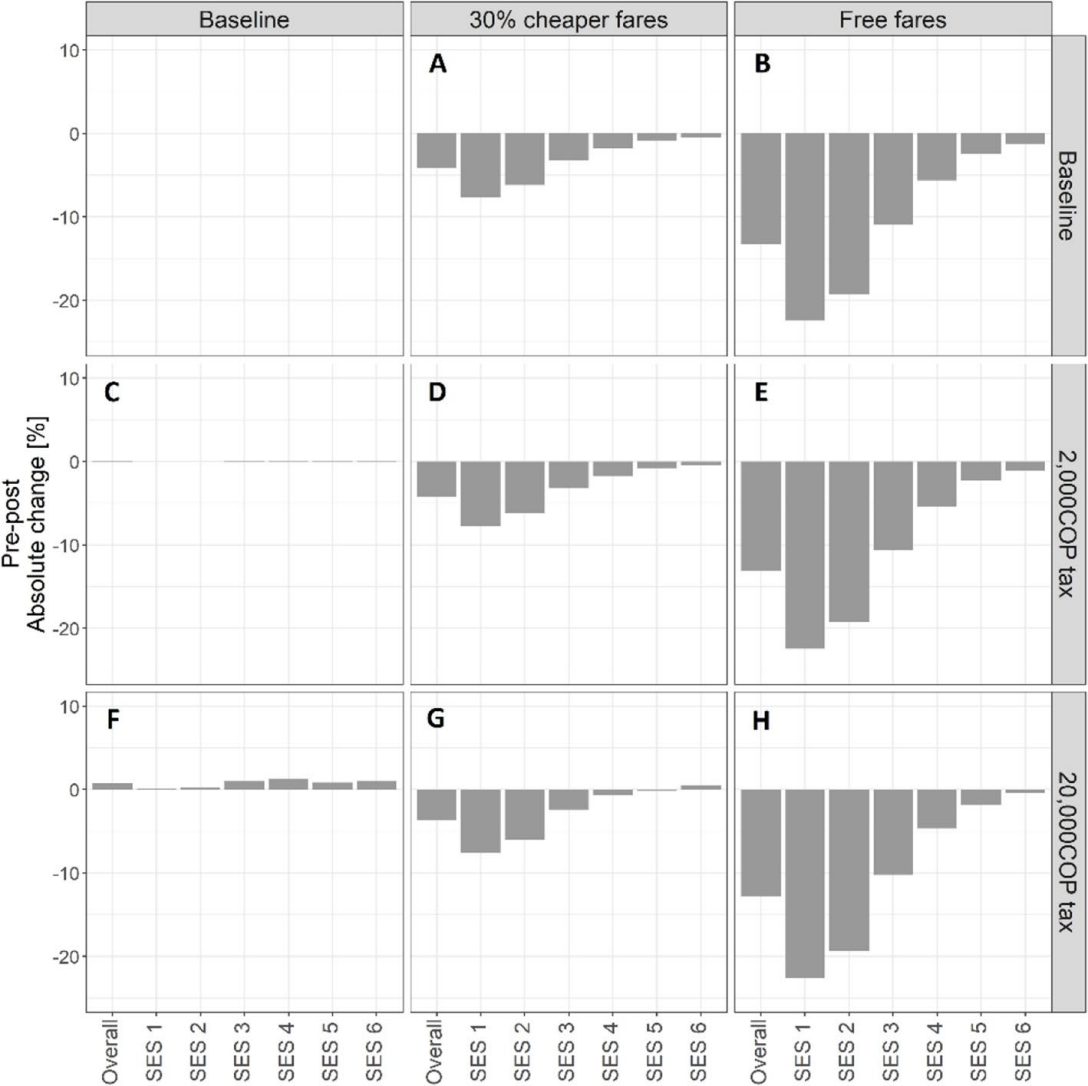
Absolute change in percent commuters meeting weekly recommended physical activity guidelines, overall and by SES (x-axis) following the implementation of **A** a 30% fare subsidy only; **B** free bus and BRT travel for all, a congestion tax of 2,000 COP only (**C**) and 20,000 COP only (**F**), and a combination of these fare subsidies and congestion taxes (**D**, **E**, **G**, **H**)

The fare policies reduced the proportion of people meeting weekly physical activity recommendations through transportation alone [3], particularly among SES1-3. The reductions observed under the 30% fare subsidy were sizable (Fig. 6A), while even larger reductions were observed in the free fare scenario, particularly among SES1-2 (Fig. 6B).

#### Congestion taxes

Congestion taxes had no influence on the prevalence of meeting weekly physical activity guidelines solely through transportation, overall and by SES (Fig. 6C & F).

#### Combination policies

The combined policies did not change the proportion of the population meeting physical activity recommendations through transportation beyond the levels observed with each fare policy alone (Fig. 6D-E & G-H).

## Discussion

We developed and calibrated an ABM which enabled a policy-oriented exploration of commuting patterns in an LMIC urban setting, characterized by high levels of necessity-based walking and inequality. Grounded in the unique context of Bogotá, Colombia, our systems approach to understanding and promoting physical activity in LMICs directly addresses recent calls within the field [14, 54]. Moreover, while our model integrated factors normatively represented in transport-related decision-making models, such as spatial accessibility and the affordability of varying forms of transportation, our model uniquely attended to other system-level influences that feature within commuters’ decision-making process, such as personal safety from crime and peer influences.

Overall, our simulation models indicated that reductions in car use and increased public transportation ridership among high SES groups can be achieved through congestion taxes. Fare subsidies on the other hand, could play an important role in promoting equitable access to public transportation among low SES commuters, thereby reducing long walking trips likely driven by economic necessity. We also observed an additive effect when combining the fare subsidy and congestion tax scenarios, highlighting their potential in achieving multiple co-benefits, including mode shifts to active transportation, reductions in travel time, and the promotion of physical activity, as reflected in the attainment of the WHO recommendations across SES.

Policies seeking to shift mode share patterns to include a higher share of active modes of transportation such as walking, bicycling and public transportation use have been recognized as important levers for health promotion. In our exploration of various fare subsidy scenarios, we observed the largest increases in public transportation use among low SES commuters who, prior to the policy, engaged in long necessity-driven walking trips. By increasing the affordability of public transportation, these low SES commuters were afforded an opportunity to shift from walking to BRT and bus use. These findings are consistent with research conducted in European cities, where increases in public transportation following the introduction of fare subsidies/ free fares arose primarily from people previously bicycling or walking [55]. Similar trends were also observed in a LMIC context [56]. In their paper, Guzman and Hessel [56] examined a 32% fare subsidy made available to the poorest residents in Bogotá. Using a regression discontinuity design, over a two-year period Guzman and Hessel [56] reported that the fare subsidy resulted in significant increases in weekday and weekend public transportation ridership among low SES individuals; a finding in keeping with patterns observed in our model.

Broadly, our simulations indicate that fare subsidies have little to no impact on mode share among mid to high SES commuters, nor are they effective in reducing private vehicle use. This finding is consistent with the available evidence. Fearnley for example, note that free fares have little to no impact on promoting substitutions from car to public transportation use [55]. A review of interventions with demonstrated effectiveness in reducing car use, suggests that fare subsidies may need to be implemented in combination with other strategies to effect car use [57]. We did, however, observe that congestion taxes resulted in modest reductions in car use through shifts from car to bus and BRT use, primarily among mid-to-high SES groups. While there is limited robust evidence regarding the influence of congestion pricing schemes on the transition to more active modes of transportationt [58] our findings appear to align with the findings of those few available studies. For example, in the review by Kuss and Nichols, who synthesized studies centered on cities within Europe and the United Kingdom, decreases in car use ranging from 12-to- 33% were reported in zones where private vehicles were charged a congestion tax [57].

Our model allowed us to explore whether mode shifts impacted total and active travel times. For fare subsidies, our simulations indicated little to no change in the total and active median travel times overall or by SES, for almost all modes. A recent systematic review of 27 studies reported that public transport users accumulated an additional 8–33 min of walking each day because of their commute [59]. However, the review was limited largely to high income city contexts where walking prevalence is low. In contexts where necessity-based walking is high, we observed that fare policies resulted in significant reductions in the median walking times among those engaging in walking only trips, specifically low SES commuters who had walked for long periods prior to the implementation of the policy. In Bogotá, socioeconomically disadvantaged populations are disproportionately represented at the periphery of the city; a distribution that sees their economic disadvantage compounded by structural constraints to accessing public transportation in a timely and efficient manner. As such, our simulated findings indicate that fare policies could play an important role in addressing time scarcity by inducing a shift from walking to public transportation. This is relevant because time scarcity may not only limit activities critical to health and wellbeing, but which itself has been directly linked to poorer mental health outcomes [16].

In our model, congestion taxes had no impact on active and total travel time for all modes, overall or by SES. Consistent with these results, Nakamura et al. [60] found that the London Congestion Charge only increased active travel by about three min. However, Nakamura et al. also found moderate positive impacts on walking and bicycling among car users and low SES households (N﻿akamura et al. [60]). We found little to no impact on the lowest SES groups, and only modest positive impacts on public transportation use among mid-to-high SES commuters. Differences between our results and those of Nakamura et al. likely reflect very different contexts in high-income countries compared to LMICs where car ownership is very low, and walking is already very high among low SES groups [61]. In addition, issues of personal and road traffic safety may reduce the attractiveness of walking and bicycling as an alternative form of transportation for higher SES individuals in LMICs. Considered together, these findings support the use of congestion taxes as policy levers capable of achieving substitutions from car use to more active modes of transportation, without increasing commuting times.

We also explored how fare subsidies and congestion taxes impacted the attainment of the WHO weekly physical recommendations. We found that congestion taxes do not impact the proportion of the population attaining weekly physical activity through transportation alone. These findings align with patterns observed in Stockholm, Sweden where no significant effects on physical activity were observed among local residents relative to two other comparison cities which did not have congestion pricing [62]. In our simulation of fare policies however, we observed a relatively large reduction in the proportion of residents meeting recommended levels of physical activity through transportation, particularly those in the low SES group. These findings are not all that surprising in a LMIC city like Bogotá, where a high proportion of the population ordinarily meet WHO physical activity guidelines from walking only trips [61]. While reductions in walking for transportation are broadly considered undesirable given their health benefits, concerns arise about the ethics of encouraging behaviors driven by circumstances beyond individual choice. This is particularly relevant for walking only trips that are very long (≥ 60 min one-way) and necessitated by unequal access to jobs, affordable housing, and reliable, and affordable public transportation. In these contexts, promoting walking as a form of physical activity can be seen as capitalizing on existing inequities rather than truly promoting healthy choices. On the other hand, reducing long walking trips can increase discretionary time available for other health-promoting behaviors including leisure time physical activity and healthy meal preparation [14]. Our findings therefore suggest that fare subsidies may result in important trade-offs that can play an important role in promoting equitable access to transportation and in addressing time scarcity through the reduction of long necessity-based walking trips in Latin America, where public transportation expenditure can exceed 25% of low-income households’ total expenditure [14, 15].

An important strength of our approach includes the use of an adaptable and flexible modelling framework that captures several key influences that are often not considered in transportation models including the effects of prior experiences, social influences, and safety considerations. Informed by input from local stakeholders [8] and real world data relevant to the LMIC context (including existing high levels of walking and safety issues as important concerns), the calibrated model successfully reproduced city-level mode share patterns across six different transportation alternatives and achieved modest alignment with SES-level travel patterns. The findings of the model were presented back to local policymakers and stakeholders in-person and virtually [63]. These groups had actively been engaged in identifying additional policy scenarios that the model could explore to support timely policymaking in Bogotá. Also, the agent-based modeling framework developed as part of this paper could be adapted and used to support the dynamic exploration of mechanisms and policies in other cities in the region.

The findings of this model must be considered with a few limitations in mind. The model focuses on single-mode trips and does not consider carpooling or ridesharing. We made several simplifying assumptions relating to how social influences operate and how perceptions of safety impact transportation decisions. Given the limited flexibility of Netlogo, model calibration was conducted by manually co-varying unknown parameters instead of using an established optimization process. While the calibrated model provides a reasonably good fit to the survey data overall, it overestimates bus use among the lowest SES groups while underestimating car use among the higher SES strata. These factors should be considered in the interpretation of policy effects by SES, particularly in relation to estimates of bus and car use. More broadly, our model was not designed to provide specific predictions of mode share prevalence and walking times, but to qualitatively contrast the plausible effects of different interventions overall and by SES. We also did not consider the downstream consequences of the congestion taxes, including likely reductions in congestion and associated increases in travel speeds for other motorized forms of travel such as buses and motorbikes. The estimates simulated by the model therefore are relatively conservative. The utility function captures many important elements, but others may be missing. Additional data on mode-specific crime prevalence and factors that uniquely impact bicycle use, such as considerations of weather and road traffic safety would help refine the model and provide a more nuanced representation of resident’s decision-making process and travel patterns.

## Conclusion

Commuting decisions are a result of a complex set of interactions among factors at multiple levels, including a city’s social fabric, the structuration of people and services, the location of workplaces, as well as person-level factors, such as financial means and safety. By adopting a systems-oriented approach which represents locally relevant factors in the decision-making process, this ABM examined the role of fare policies and congestion taxes on three key outcomes in a stylized LMIC city: mode share, travel time and physical activity. We observed that fare subsidies and congestion taxes are variously required to achieve reductions in car use and the promotion of public transportation across the socioeconomic spectrum. Our modelling also highlights the importance of fare subsidies in LMICs as a means of promoting equitable access to transportation among the poorest residents of a city, thereby reducing the proportion of very long walking journeys (≥ 60 min one-way) undertaken due to economic constraints. Transportation policies should be focused on maintaining participation in active travel while improving the conditions under which it occurs. In doing so our research underscores the importance of prioritizing social equity while recognizing the limitations of a choice-based model of physical activity. It also highlights the utility of a systems-lens that attends to the structuration of transportation, physical activity and therein health.

### Supplementary Information

**Supplementary Material 1.**

## Data Availability

The datasets used and/or analysed during the current study are available from the corresponding author on reasonable request.

## Abbreviations

ABM: Agent-based model
PA: Physical activity
SES: Socioeconomic strata
PT: Public transportation
LMICs: Lower-and middle-income countries
BRT: Bus rapid transit
CBD: Central business district
SISBEN: Sistema de Selección de Beneficarios
COP: Colombian pesos
WHO: World Health Organization
