# Supplementary material for: Uncovering physical activity trade-offs in transportation policy: A spatial agent-based model of Bogotá, Colombia
Source: Int J Behav Nutr Phys Act. 2024 May 8;21:54. doi: 10.1186/s12966-024-01570-1 (PMC11077730; doi:10.1186/s12966-024-01570-1)
Supplement: Supplementary file 1 — Supplementary Material 1. [file 12966_2024_1570_MOESM1_ESM.docx]

# Supplementary Material

## Supplement 1: Agent properties and actions

### S1a) Estimating personal daily income distributions by SES

To estimate the daily personal income distributions of the overall population and for each SES stratum we used data from the 2019 Bogotá Household Travel Survey [1] (see Table 1). The ‘no response’ category was ignored.

| **Table 1: Population frequency of monthly household income (COP/month) overall and by SES stratum, reported in the 2019 Bogotá Household Travel Survey [1]** | | | | | | | | | |
| --- | --- | --- | --- | --- | --- | --- | --- | --- | --- |
| **Income bins**  **(COP/month)** | | **Income bin midpoint** | **SES strata** | | | | | | **Population Total** |
|  |  |  | *1* | *2* | *3* | *4* | *5* | *6* |  |
| 1 | $0 - $828,116 | $414,058 | 2136 | 3896 | 2226 | 260 | 19 | 28 | 8565 |
| 2 | $828,117 - $1,500,000 | $1,164,059 | 1176 | 3313 | 2775 | 384 | 16 | 37 | 7702 |
| 3 | $1,500,001 - $2,000,000 | $1,750,001 | 409 | 1638 | 1944 | 384 | 43 | 22 | 4441 |
| 4 | $2,000,001 - $2,500,000 | $2,250,001 | 170 | 971 | 1737 | 488 | 74 | 33 | 3472 |
| 5 | $2,500,001 - $3,500,000 | $3,000,001 | 170 | 777 | 1885 | 859 | 123 | 60 | 3874 |
| 6 | $3,500,001 - $4,900,000 | $4,200,001 | 55 | 425 | 1544 | 989 | 167 | 78 | 3257 |
| 7 | $4,900,001 - $6,800,000 | $5,850,001 | 46 | 194 | 891 | 963 | 298 | 146 | 2537 |
| 8 | $6,800,001 - $9,000,000 | $7,900,001 | 32 | 97 | 386 | 605 | 244 | 218 | 1582 |
| 9 | > $9,000,000 | $\mu_{TB}$ or ${x͂}_{TB}$ | 23 | 49 | 237 | 670 | 386 | 480 | 1845 |
| 10 | No response |  | 381 | 765 | 1217 | 904 | 232 | 286 | 3786 |

To characterize the income distributions for these grouped data we made a series of assumptions:

1. We assumed that all observations within a given income group were clustered at the midpoint of the income bin, such that the mean of the observations in a given bin is equal to its midpoint (e.g., that the mean of the observations in the $0-$828,116 bin was at its midpoint i.e., $414,058). See ‘Midpoints’ column in Table 1.
2. Given that the midpoint for the open-ended top interval cannot be estimated using the approach outlined in 1, we used a commonly applied estimator (which assumes that the incomes in the top bin are Pareto distributed) to estimate the mean and median income of the top income group [2], where the smaller of the two estimates is considered more accurate and therefore used as the midpoint. The median ${(x͂}_{TB})$ and mean ${(\mu}_{TB})$ were calculated using the equations below.

$$v=\frac{c-d}{b-a}$$

and,

${x͂}_{TB}={10}^{\left( \frac{0.301}{v} \right)}*X$ or $\mu_{TB}=X*\left( \frac{v}{v-1} \right)$

Where,

$c=$ log(frequency in highest income bin + frequency in 2^nd^ highest income bin)

$d=$ log(frequency in 2^nd^ highest income bin)

$b=$ log(lower limit of highest income bin)

$a=$ log(lower limit of 2^nd^ highest income bin)

$X=$ lower limit of the highest income category

For example, using data from the survey (Table A1), the average income of the top income bin for SES 1 was calculated as:

$x_{TB}={10}^{\left( \frac{0.301}{1.4465} \right)}*9,000,000=12,905,526$ || $\mu_{TB}=9,000,000*\left( \frac{1.4465}{1.4465-1} \right)=18,751,713$

The midpoint of the top income bin was calculated uniquely for each SES stratum because the frequency of observations in the highest two income bins (which are used in the above calculation) differs by SES (see Table 2). Since the median estimate was consistently the smaller of the two estimates, we used the median as the midpoint estimate for all SES strata. The discrepancies in the estimates (e.g., midpoint of highest income bin for SES 2 is higher than midpoint for SES 6) can be explained by variation in the frequency of observations in each income bin, by SES, which affect the magnitude of the estimate in each SES stratum.

| **Table 2: Income midpoints estimated for the highest (open-ended) income bin for each SES stratum (in Colombian Pesos per month)** | | | | | | |
| --- | --- | --- | --- | --- | --- | --- |
|  | **SES strata** | | | | | |
|  | *1* | *2* | *3* | *4* | *5* | *6* |
| **Income midpoint of highest income bin (COP/month)** | $12,905,526 | $14,532,041 | $13,494,959 | $11,679,217 | $11,042,326 | $10,634,195 |

We then converted the midpoint of the monthly household incomes to daily incomes by dividing by 30. Further, we estimated the personal daily income by dividing the daily household income by 3, which is the average household size in the 2019 Bogotá Household Travel Survey. Using these daily personal income midpoints for each income bin and the frequency of observations, we explored the fit of different types of distributions supported by Netlogo, including normal, log normal and gamma distributions using the fitdistrplus package v. 1.1-3 [3] in R [4]. Using this package, we estimated the mean and standard deviation of the best-fitting function for each of the six SES strata. Ultimately, SES 1 and 2 were modelled using a log normal distribution while SES 3 to 6 were modelled using normal distributions. Table 3 reports the mean and standard deviation of the daily personal income distribution, by SES, in Colombian Pesos (COP) per day.

| **Table 3: Daily personal income distribution parameters for each SES stratum (in Colombian Pesos per day [COP/day])** | | | | | | | |
| --- | --- | --- | --- | --- | --- | --- | --- |
| **Parameter (COP/day)** |  | **SES stratum** | | | | | |
|  |  | *1* | *2* | *3* | *4* | *5* | *6* |
| LogMean |  | 9.1104 | 9.3958 |  |  |  |  |
| LogSD |  | 0.7698 | 0.8053 |  |  |  |  |
| Mean |  | 12,168 | 16,648 | 28,593 | 54,135 | 75,198 | 84,248 |
| Standard deviation |  | 10,937 | 15,903 | 25,294 | 35,911 | 35,931 | 36,255 |

### S1b) Safety risk rule

To determine this probability, each agent $i$first estimates the perceived risk to personal safety score${sr}_{im}$ associated with travelling with each mode $m$ in their choice set. They do this by averaging: 1. the mean of their own past experiences of crime/ victimization using mode $m$, and 2. the average experiences of all their social contacts using mode $m$. The resulting safety risk score of each mode ${sr}_{im}$ is multiplied by the agent’s safety risk sensitivity $s_{i}$ and the product is subtracted from one to determine the probability of keeping any given mode in the agent’s choice set $p_{im}$. This probability is updated each day and is described by the following function:

$$p_{im}=1-(s_{i} \times{sr}_{im})$$

### S1c) Mode choice rule

Before the utility of each mode is evaluated, agents consider the relative cost of travel via each mode. If the relative cost (including fuel and parking costs as appropriate) of this mode, divided by the agent’s daily personal income, is greater than one (i.e., the cost of travel exceeds the agent’s daily personal income), then the mode is assumed an ‘unaffordable’ option and it is excluded from the agent’s choice set. If, however, the mode is deemed ‘affordable’ its utility is calculated by considering the out-of-pocket, travel time and exercise cost of travel via that mode.

In our model, the utility function is expressed in the form:


$$U_{im}(t+1)=mval*\frac{C_{m}}{w_{i}}+tval*\frac{\left( \frac{w_{i}}{8}*\frac{{ett}_{im}(t)}{60} \right)}{w_{i}}+exval*\frac{{actt}_{im}(t)}{{ett}_{im}(t)}*{sl}_{i}$$

Out-of-pocket & travel time cost

Exercise cost

Where, the utility $U_{im}(t+1)$ of any given mode$m$, as perceived by agent$i$, is modelled as a function of the out-of-pocket, travel time and exercise cost of travel. These include the daily cost $C_{m}$ of travel (including fuel and parking costs as appropriate), as well as the estimated total travel time (${ett}_{im}(t)$) at time $t$, which is operationalized as a monetary cost by multiplying the number of hours travelled by agent$i's$ hourly income $w_{i}$. The coefficients ${tval}_{i}$and ${mval}_{i}$respectively represent the global value of time (specifically, time not devoted to exercise) and money. The exercise cost factors in the active travel time ${actt}_{im}(t)$, which is expressed as a fraction of the overall total travel time (${ett}_{im}(t)$), the value of exercise ($exval$) and the slope of the terrain. For car and motorbike trips the slope ${sl}_{i}=1$ as it does not influence the attractiveness of either mode given that these two modes do not involve any active travel.

The estimated travel time (${ett}_{im}(t)$) of for agent $i$ using mode $m$ is evaluated by considering the average of the time the agent estimates it will take them to travel to work using the chosen mode. This includes the access time ${at}_{im}(t)$ (e.g., the total time taken to walk from home to the chosen bus stop and from the bus stop to work), the passive portion of the commute ${cett}_{im}(t)$ (e.g., from the time they step on the bus to the time they disembark) as well as any waiting time ${wt}_{mi}(t)$ involved (e.g., the time spent waiting for the bus to arrive). Agents also consider their own average travel time over the course of their past 20 trips using $m$ ${avgTlog}_{im}(t)$ as well as the average time their friends/ social contacts $k$ spent travelling via that mode over the course of the past 20 trips ${avTlog}_{im}(t)$. As such, the estimated travel time is expressed by the function:

$${ett}_{im}(t)= \frac{1}{3}\left( \left[ {at}_{im}(t)+{wt}_{im}(t)+{cett}_{im}(t) \right]+{avTlog}_{im}(t) +\frac{1}{N_{k}}\sum_{k}^{N_{k}} {avTlog}_{km}(t) \right)$$

### S1d) Memory formation

Memories of commute time are stored in a travel time log ${Tlog}_{im}$ which is unique to each mode $m$. The log captures 20 days of most recent travel times for each mode, whereby the oldest experiences are forgotten as new ones are formed. Memories of crime/ victimization are stored in a crime log ${SRlog}_{im}$ specific to each travel day using mode $m$. Each day, the crime log records a one if the agent is victimized during their travels using mode $m$, and a zero if no crime/ victimization takes place. The prevalence of crime and therefore the probability of being victimized while commuting using a particular mode is informed by data (Supplement S2e). Each agent’s crime log captures 120 days of most recent travel experiences using mode $m$; the oldest experiences are forgotten as new ones are formed.

## Supplement 2: Spatial characteristics of the model

### S2a) Calculating the area occupied by each SES

Colombia’s national classification system uses a socioeconomic strata (SES) scale to classify households based on the physical characteristics of dwellings and the surrounding neighbourhoods ranging from SES1 (lowest) to SES6 (highest). The system was developed to enable better targeted public service tariffs whereby subsidies are given to the lowest SES households and higher tariffs are paid by the highest SES groups. The SES classification system broadly serves as a proxy for household income.

Using the Group Stats plugin in QGIS [5] and 2014 block level SES data from the city of Bogota [6], we calculate the area, in square meters, occupied by each SES stratum in the city of Bogota (see Table 4). These areas were then expressed as a percentage of the total area of the city. We used these data along with the map of Bogota to characterise, in an abstract way, the spatial distribution and extent of each SES stratum in the model. Figure 1 shows how the City of Bogota and the distribution of SES strata throughout the city were represented in the ABM.

| **Table 4: Area occupied by each SES stratum in Bogota** | | |
| --- | --- | --- |
| **SES stratum** | **Area (m^2^)** | **% of total city area** |
| 1 | 12883373.66 | 8 |
| 2 | 50261682.45 | 30 |
| 3 | 61771052.5 | 37 |
| 4 | 21366431.04 | 13 |
| 5 | 10780083.97 | 6 |
| 6 | 10985296.68 | 7 |
| Total city extent: 168047920.3 m^2^ = 168.05 km^2^ | | |

| 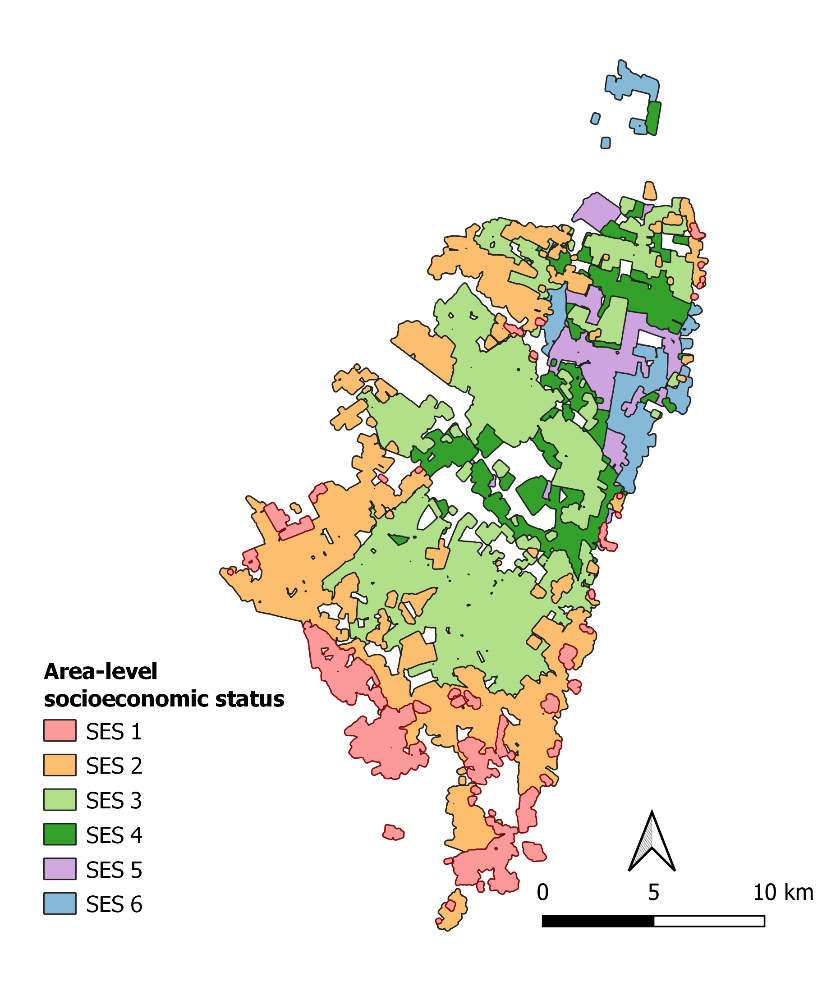 | **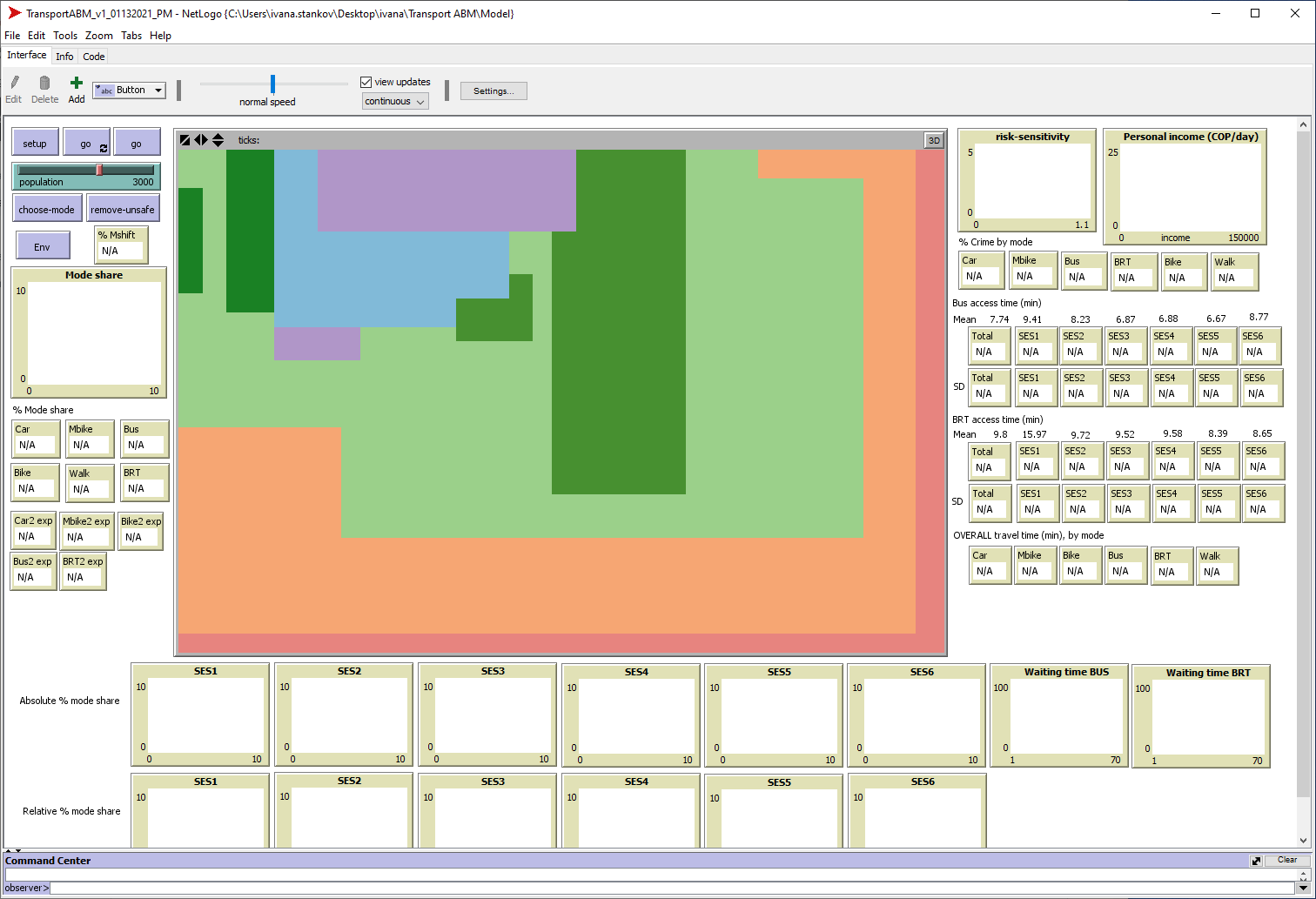** |
| --- | --- |
| **Figure 1:** Map of the City of Bogota along with the SES strata and their distribution throughout the city (*left*) and the abstract representation of the city in the ABM environment (*right*) | |

### S2b) Number of people in each SES stratum

We used data from the 2019 Bogota Household Travel Survey to inform the percentage of the population belonging to each SES stratum [1].

| **Table 5:** **Percent population by SES** | |
| --- | --- |
| SES stratum | % of the population |
| 1 | 11 |
| 2 | 30 |
| 3 | 36 |
| 4 | 16 |
| 5 | 4 |
| 6 | 3 |

### S2c) Defining the work environment by SES

| **** | | **Figure 2:** Bogota job density from Guzman & Oviedo (2018) [7]  Based on the map and the spatial distribution of the six SES zones, we assumed that the first job density cateogry in the figure corresponded to SES 1 and 2 zones. The second density category was assumed to correspond to SES3, while the third corresponded to SES 4-6, and the final category corresponded to the CBD, or ‘workzone’ in the model.  We estimated the upper and lower bounds of the number of jobs in each zone by multiplying the densities by the corresponding areas. We used the midpoint of this range to approximate the number of jobs thorughout the city (N = 2,336,958).  We further scaled down this number so that each workplace in the model, represented 50 workplaces in the real world. Thus the total number of workplaces in the model was initalized as N = 46,739. We did this due to run-time constraints imposed by an already large agent population. Overall, the number of workplaces were specified as follows; Central Business District: n=16495; SES 1 & 2: n=3705; SES 3: n=11351; SES 4, 5 & 6 n=15188. |
| --- | --- | --- |
| 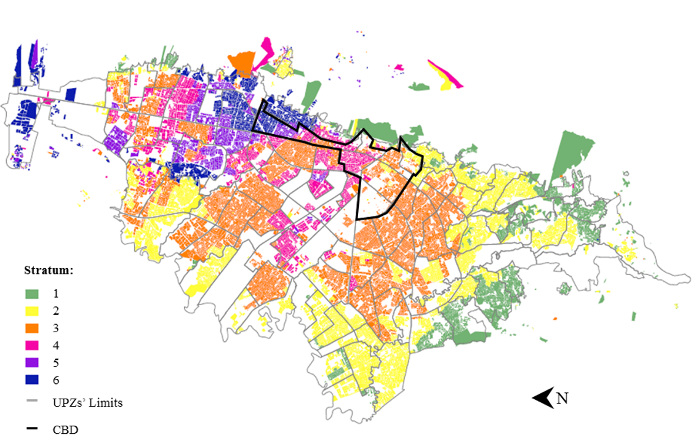 | **Figure 3:** Map depicting the location of the central business district (CBD) in relation to the area-level socioeconomic strata in Bogota [8]  This figure was used to inform the positioning of the central business district in the model. | |
| ****  **Figure 4:** Maps of work trip destinations by income level  This map and information published by Oviedo and Guzman [9] were used to inform how workplace destinations were assigned to agents, based on their level of income. The rows below show how these different zones were represented in the model and the proportion of agents that were assigned to workplaces in each of these zones. Given that income groups depicted in this figure are not defined within the chapter, we assume that authors define low-, middle- and high-income levels as expressed by Cantillo-García et al. [10]. That is, low income as <1.3mill COP/mth (~ <14,444COP/day), middle income (1.3-4mil COP /mth ~ 14,445 – 44,444 COP/day) and high income (> 4mil COP/mth ~ 44,445 COP/day), where the daily figures were calculated by adjusting for the average household size (i.e., n = 3) and dividing by 30. | | |

| **A.**  **Workzone (core CBD in red) + High income area = expanded CBD (black area)**:  90% of high income agents work in the expanded CBD, and the rest work outside of these areas  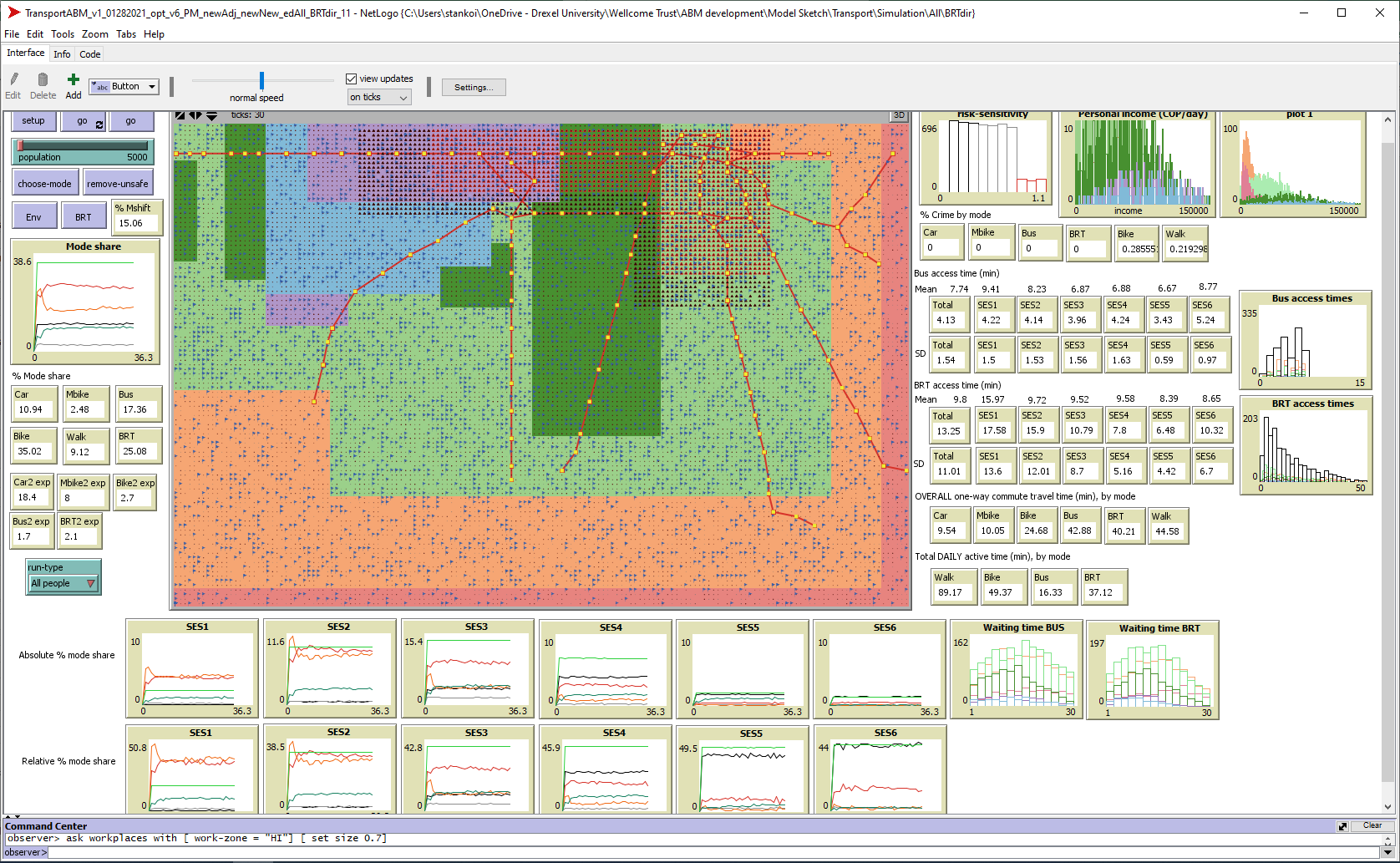 | | **Work trip destinations of high-income residents** |
| --- | --- | --- |
| **B.**  **Work trip destiantions CBD (black and red area):**  77% of middle income agents work in the expanded CBD  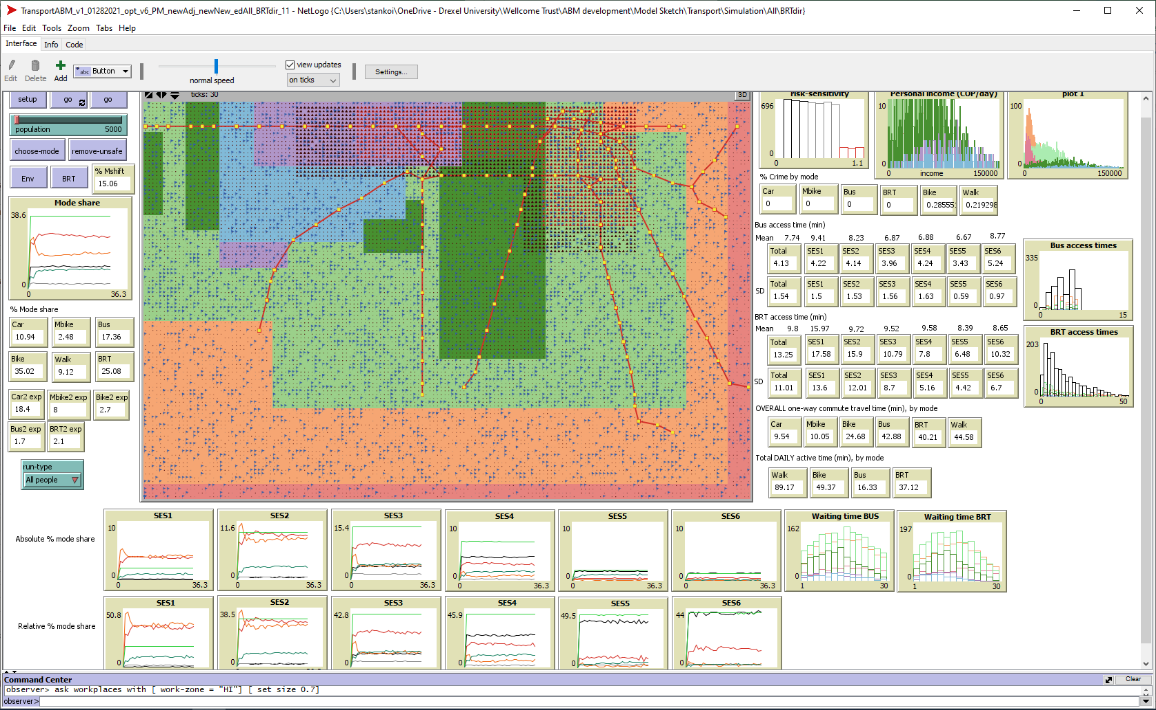 | **Middle income zone (black area extending out from the expanded CBD zone)**:  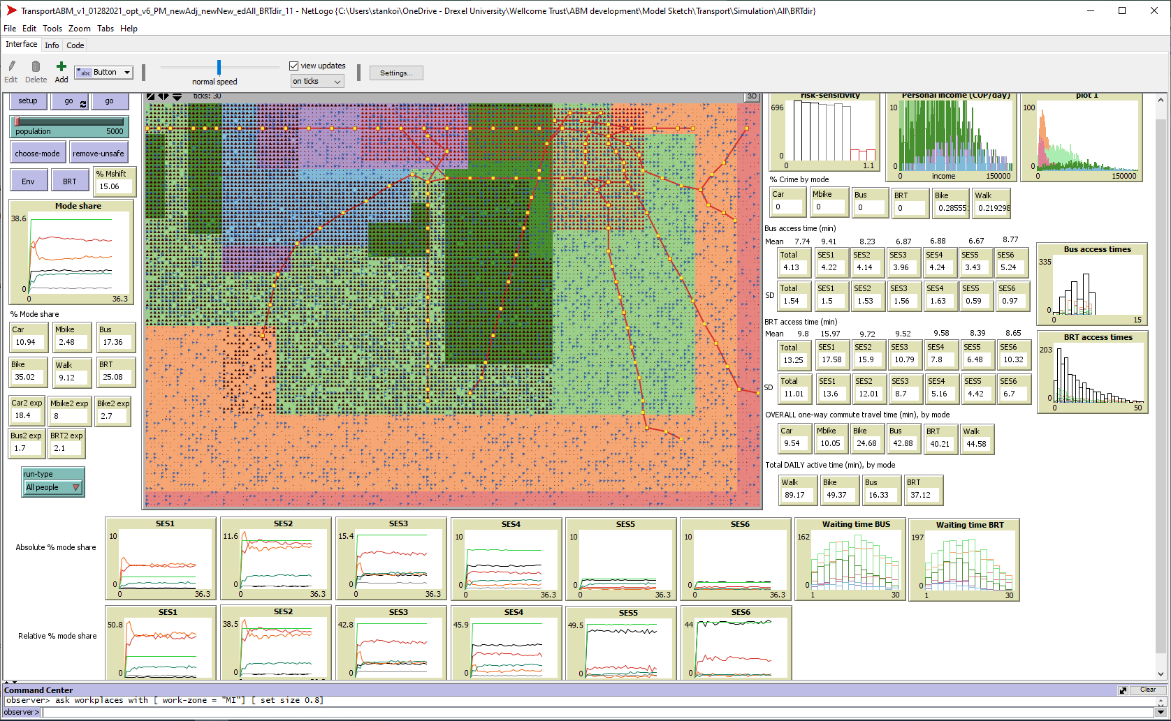12% of middle income agents work in the middle income area, and the rest (12%) work in other areas | **Work trip destinations of middle-income residents** |
| **C.**  **Work locations of people with low income:** 28% of low income agents work in CBD (red),  the rest work across the remainder of the city.  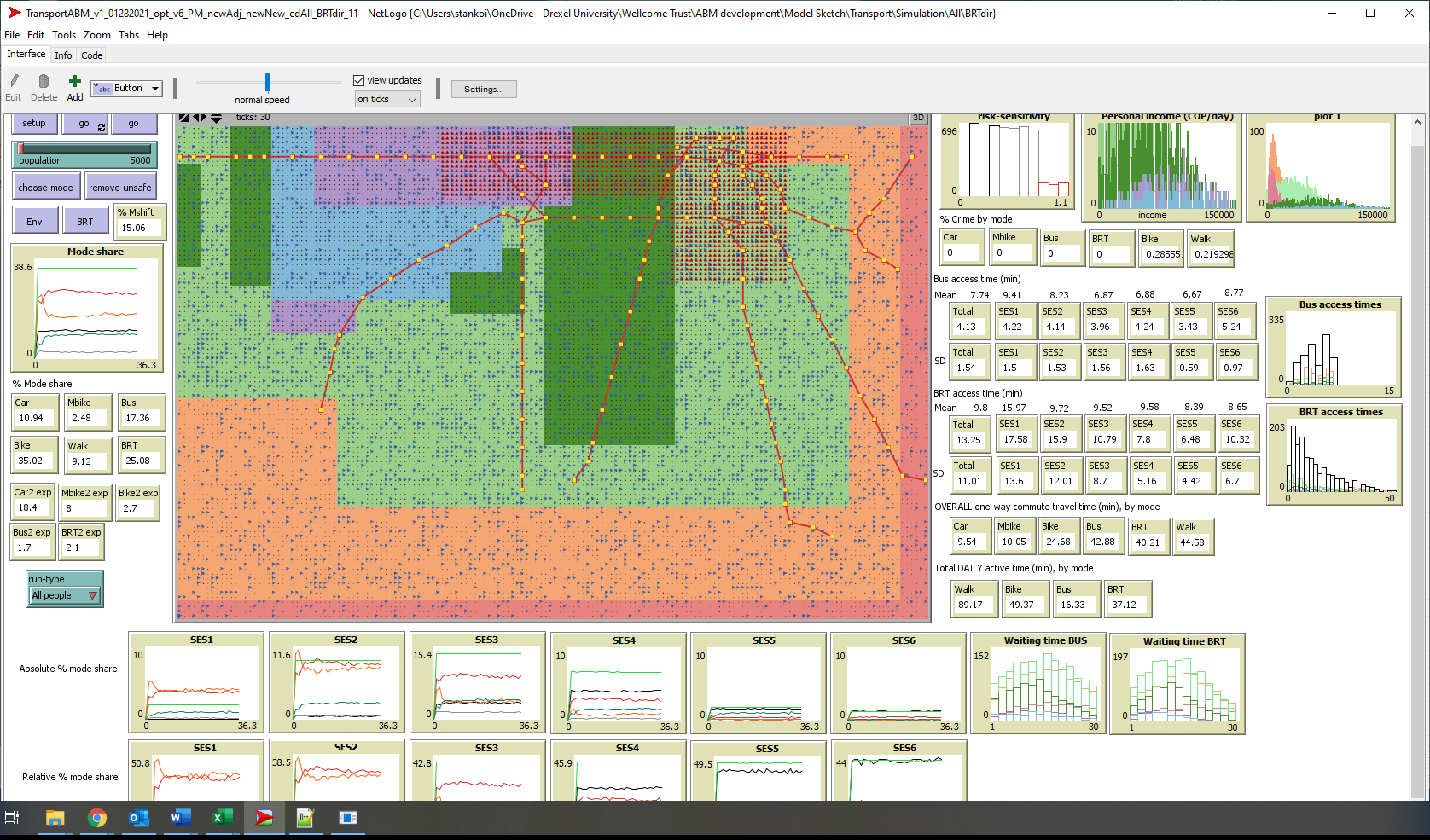 | | **Work trip destinations of low-income residents** |

**Figure 5:** Shows how the work trip destination by income level from Oviedo and Guzman [9] (right column) were operationalised in the ABM (left column), including **A**. for high income, **B.** middle income, and **C.** low income earners.

### S2d) Slope penalty

Research by Meeder et al. suggests that for every 1% increase in slope, the attractiveness of walking decreases by 10% [11]. The effect of slope, therefore, will be accounted for in the model using the effect size modifier ${sl}_{w}$. The topography of Bogota city was explored in Google Maps. The city is fairly flat, except for the central business district, which is situated at an elevation of some 25m to 40m above the surrounding areas. To approximate the average slope, two sets of points, one from the central business district (point A) and two from different locations (points B and D) in the surrounding areas were examined.

The first pair of points was 3800m apart, with central business (point A) situated at an elevation of 25m above the surrounding area (point B). The slope was calculated by dividing the rise (elevation) by the run (horizontal distance) which was estimated at 0.66%.

Elevation = 25m

Distance = 3800m

**A**

**B**

$$\% slope=\frac{25}{3800}*100$$

$=0.66\%$

The second pair of points was 10,300m apart, with central business (point A) situated at an elevation of 40m above the surrounding area (point D). The % slope for points A and D was estimated at 0.39%.

Elevation = 40m

Distance = 10,300m

**A**

**D**

$$\% slope=\frac{40}{10300}$$

$=0.39\%$

Using these two estimates, we calculated the average slope:

$$Avg \% slope= \frac{0.66+0.39}{2}=0.52\%$$

Given these two pairs of slope estimates, we assumed a 0.5% increase in slope from surrounding city areas (points B and D) to the central business district (point A). According to Meeder et al.’s study [11], this translates to a 5% decrease in the attractiveness in walking. Therefore, an additional marginal cost for walking on sloped terrain was featured in the walking utility model for those travelling from peripheral areas to/from the central business district (${sl}_{w}=1.05$). On the other hand, agent’s commuting within the central business or within surrounding areas were not imposed a slope penalty (${sl}_{w}=1$).

Slope is also an important consideration in determining the perceived attractiveness of bicycling. According to research by Rodriguez and Joo cyclists are around 2.5 times more sensitive to slope than pedestrians [12]. The findings by Meeder et al. and Rodriguez and Joo are considered together to estimate the impact of slope on the overall attractiveness of cycling [11, 12]. In our model therefore, for every 1% increase in slope, the attractiveness of bicycling decreases by 25%. Since the maximum slope increase estimated in our model is 0.5% for those commuting between the central business district and peripheral areas, the attractiveness of bicycling is 12.5% less than those not traversing sloping terrain as part of their commute. Therefore, for those commuting between the central business district and peripheral areas the utility/ cost of bicycling is 1.125 higher than for other commuters (i.e., ${sl}_{b}=1.125$).

### S2e) Estimating crime prevalence for each mode of transportation

We sourced 2015 data of crimes reported to the Bogota police department. These data were available for Transmilenio BRT (where we summed reported crimes that occurred on the Transmilenio bus feeder, Transmilenio bus and the Transmilenio station), Bus (combination of bus stop and bus) and crimes reported in the streets, which we assumed captured crimes reported by pedestrians walking. The crimes reported to police at each location range in type and include sexual crimes, homicides, robbery, personal injuries, and gender-based violence [13-17]. We aggregated across all crime types to find the total number of crimes reported on the BRT, bus and among pedestrians. To estimate the prevalence of crimes in the streets (i.e., experienced by pedestrians), we aggregated crimes reported in Bogotá, by type, across the following locations (i.e., those reported under the “Clase de sitio” variable: “CICLO RUTA”, “FRENTE A ALMACEN-VIA PUBLICA”, “FRENTE A BANCO-VIA PUBLICA” “FRENTE A COLEGIO-VIA PUBLICA”, “FRENTE A EDIFICIO-VIA PUBLICA”,” FRENTE A RESIDENCIAS-VIA PUBLICA”,” FRENTE A CLINICA U HOSPITAL-VIA PUBLICA” “SOBRE ANDEN-VIA PUBLICA”, “FRENTE CONUNTO-VIA PUBLICA”, FRENTE A EMPRESA-VIA PUBLICA”, “FRENTE IGLESIA-VIA PUBLICA”, “RODANDO SOBRE VIA-VIA PUBLICA”, “VIAS PUBLICAS”, “SEMAFORO”, “SENDERO PEATONAL”, “TRAMO DE VIA”, and” ZONAS ZUALES-VIA PUBLICA”.

We then used the 2015 total trips by mode data collected as part of the 2015 Prudencia Bogota mobility survey [18] as the denominator to calculate the prevalence of crime for BRT, bus and walking (Table 6). Similar data were not available for bicycle, car and motorcycles so we used expert consultation to estimate the crime prevalence on these modes relative to bus, brt and walking. Based on extensive discussion, consensus emerged over the fact that bicycle trips are the second most unsafe, ranking midway between walking and brt; the crime prevalence for bicycle users was therefore assumed to be 0.5% (i.e., (0.918-0.076)/2=0.5%). Car and motorcycle trips were considered the safest, with an estimated prevalence of 0.015% for car trips, and 0.01% for motorcycle trips (Table 6, purple text).

To estimate crime prevalence by gender for trips made by different modes we used data from the Prudencia Bogota mobility survey [18] which asked participants whether they have been the victims of crime. Given that the reports of crime do not reference a particular trip or the mode on which the crime occurred, we cross-tabbed the trips participants reported with the crimes recorded to estimate the proportion of participants, by gender, that reported experiencing a crime at least once for any given mode (Table 7). We used these proportions to estimate how the total prevalence rates reported to police are distributed by gender (Table 8). We did this by multiplying the total prevalence for a given mode (as derived from reports of crime to the Bogota police department) by the gender-specific prevalence for that mode in Table 7 (e.g., for female pedestrians (Table 8) = total % pedestrian crime (Table 6)* % crime reported by women on walking trips (Table 7) = 0.918%*64.2%=0.590%. The estimated crime rates by mode and gender used in the model are those featured in Table 3.

| **Table 6: Percent crime prevalence by mode*** | | | | | |
| --- | --- | --- | --- | --- | --- |
|  |  | **Female** | **Male** | **Total %** | **Data year** |
| least safe | **Pedestrian** |  |  | 0.918 | 2015 |
|  | **Bicycle** |  |  | **0.500*** |  |
|  | **BRT** |  |  | 0.076 | 2015 |
|  | **Bus** |  |  | 0.028 | 2015 |
|  | **Car** |  |  | **0.015*** |  |
| most safe | **Motorcycle** |  |  | **0.010*** |  |

*calculated by using reported crime data from the Bogota police department
and the total number of trips, by mode as the denominator

| **Table 7: Estimated share of crimes by mode and gender from the Bogota mobility survey** | | |
| --- | --- | --- |
| Proportion of people that ever-experienced crime on a given mode of transport by gender | | |
|  | men | women |
| **BRT** | 0.361 | 0.639 |
| **Bus** | 0.391 | 0.609 |
| **Car** | 0.467 | 0.533 |
| **Motorcycle** | 0.740 | 0.260 |
| **Bicycle** | 0.739 | 0.261 |
| **Pedestrian** | 0.358 | 0.642 |

| **Table 8:** **Estimated percent crime prevalence by mode*** | | | | | | |
| --- | --- | --- | --- | --- | --- | --- |
|  |  | **Female** | **Male** | **Total %** | **Total proportion** | **Data year** |
| least safe | **Pedestrian** | 0.590% | 0.328% | 0.918 | 0.0092 | 2015 |
|  | **Bicycle** | 0.130% | 0.370% | **0.500*** | 0.0050 |  |
|  | **BRT** | 0.048% | 0.027% | 0.076 | 0.0008 | 2015 |
|  | **Bus** | 0.017% | 0.011% | 0.028 | 0.0003 | 2015 |
|  | **Car** | 0.008% | 0.007% | **0.015*** | 0.0002 |  |
| most safe | **Motorcycle** | 0.003% | 0.007% | **0.010*** | 0.0001 |  |

*calculated by using reported crime data from the Bogota police department and the total number of trips,
by mode as the denominator

### S2f) Protocol evaluating the number of bus and BRT stops in each SES stratum

We used QGIS to visualize 2014 geospatial data relating to area-level SES in Bogota [6] as well as bus and BRT stations and their spatial distribution in the city [19, 20]. We then created 100-meter buffers around each SES stratum. These buffers were created to capture BRT and bus stops located on broad streets / non-residential corridors with no assigned SES. A 100-meter buffer was chosen as this was approximately the width of the widest road in Bogota. We then created a set of variables in the bus and BRT stop attribute tables that indicates which SES stratum intersects with a given stop (some stops were in narrow corridors next to residential areas that belong to three or four different SES strata). We then exported the attribute tables into excel to finalize the assignment of each bus and BRT stop to a single SES stratum.

Each bus and BRT stop which was clearly located in just one SES stratum (i.e., not in between two SES areas) was assigned to that stratum. For bus and BRT stops that were located at the intersection of two or more SES areas, the transit stop was assigned to one of the SES strata at random using the =CHOOSE(RANDBETWEEN) function in excel, which selects at random, one of the SES strata intersecting with the given transit stop.

This process identified a total of 7694 bus stops, n=584 of which were located in areas which could not be linked to any one SES stratum and were therefore excluded (see Table 9 for more information on bus stop counts by area-level SES). Moreover, of the identified n=149 BRT stops, 23 were located in areas with unknown SES and were therefore excluded (see Table 10 for more information). The bus and BRT stops located outside the city boundary of Bogota and those that could not be linked to any one SES stratum were excluded.

| **Table 9: Bus stop counts by SES** | | | | | | | |
| --- | --- | --- | --- | --- | --- | --- | --- |
|  | **SES stratum** | | | | | |  |
|  | 1 | 2 | 3 | 4 | 5 | 6 | Total bus with known SES |
| Bus stop count | 620 | 2323 | 2835 | 769 | 327 | 236 | 7110 |
| % of total | 9% | 33% | 40% | 11% | 5% | 3% |  |
| Blank | 584 |  | | | | |  |

| **Table 10: BRT stop counts by SES** | | | | | | | |
| --- | --- | --- | --- | --- | --- | --- | --- |
|  | **SES stratum** | | | | | |  |
|  | 1 | 2 | 3 | 4 | 5 | 6 | Total BRT with known SES |
| BRT stop count | 0 | 12 | 76 | 27 | 8 | 3 | 126 |
| % of total | 0 | 10% | 60% | 21% | 6% | 2% |  |
| Blank | 23 |  | | | | |  |

Ultimately, we identified 126 BRT stops in the city of Bogota and located these according to their spatial distribution within the ABM (Figure 6). We used BRT route maps to inform, as closely as possible, the service connections between BRT stops in the ABM (Figure 7). Of note, our definition of the BRT network includes the feeder buses that transport commuters from different areas of the city to the BRT stops.

| 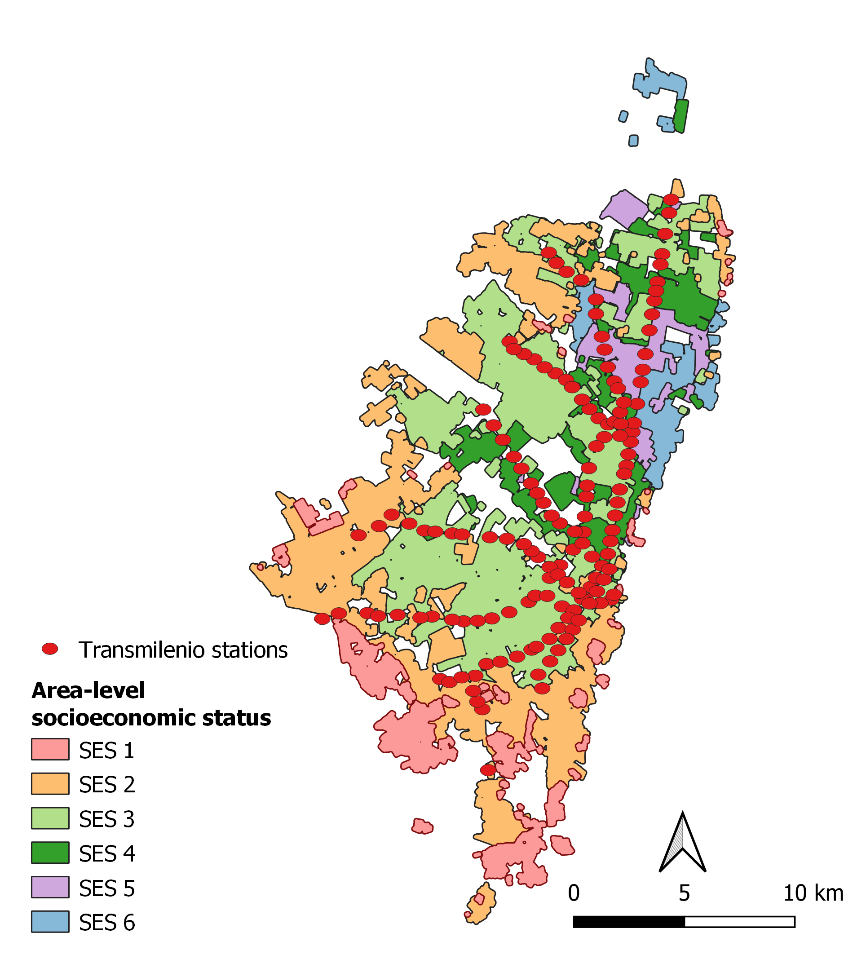 | 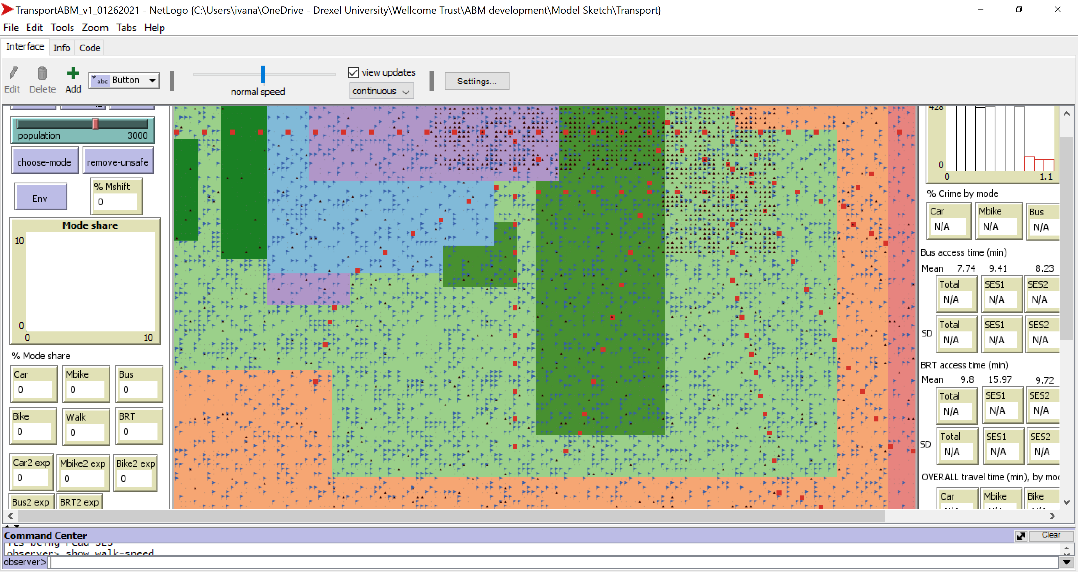 |
| --- | --- |
| **Figure 6:** Map of the City of Bogota along with the SES strata and their distribution throughout the city (*left*) and the abstract representation of the city in the ABM environment (*right*) | |

| 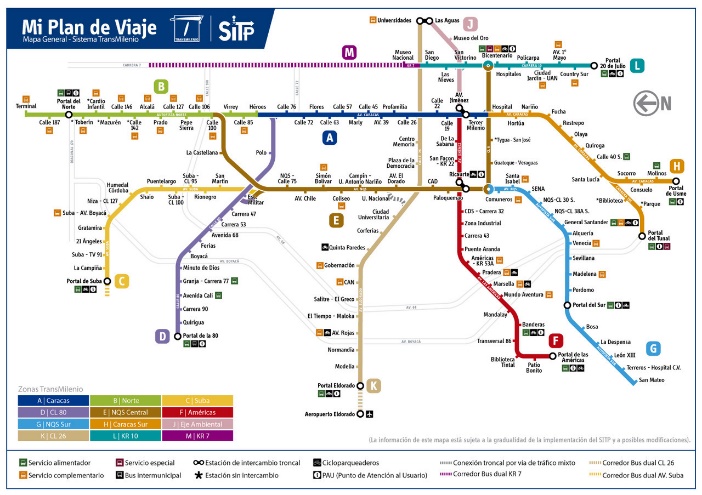 | 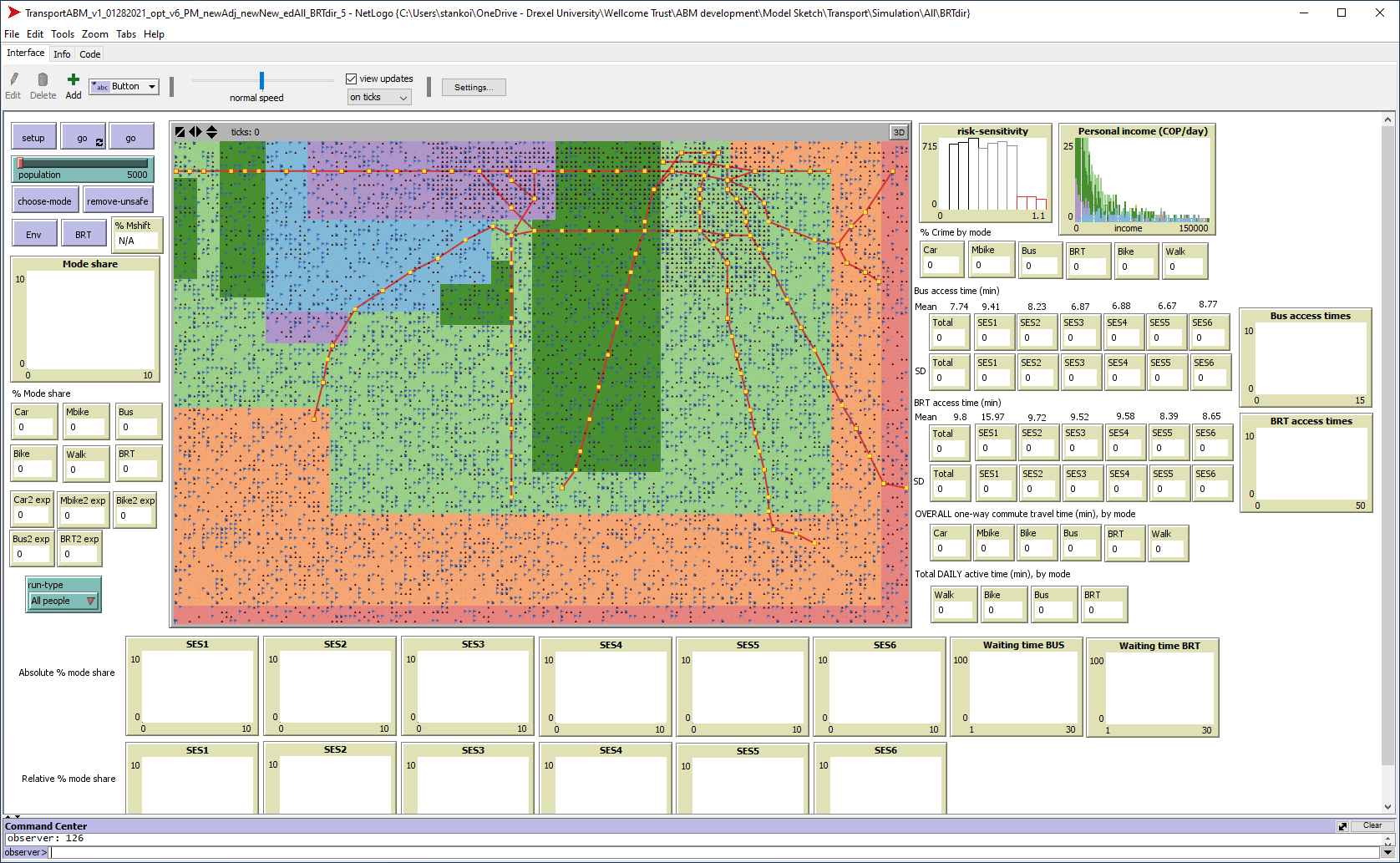 |
| --- | --- |
| **Figure 7:** Map representing the TransMilenio service network, including the feeder buses that carry passengers to BRT stops (*left*) and the representation of Bogota’s BRT network in the ABM (*right*). | |

## Supplement 3: Sensitivity analysis

A sensitivity analysis was conducted to test the model’s sensitivity to uncertainty in the specification of attributes relating to the ‘safety risk rule’, specifically, the crime prevalence and safety risk sensitivity distribution.

The safety risk rule involves a calculation of the safety risk score of each mode, which agents use to probabilistically determine whether to include a given mode in their choice set. For example, if the safety risk is very high, then the probability of including a given mode in their choice set will be lower than a mode with a comparatively lower safety risk. The calculation of the safety risk score requires consideration of the crime prevalence by mode and gender, and an agent’s safety risk sensitivity.

In the absence of mode and gender specific crime prevalence data, we used 2015 personal crime data [13-17] along with trip data [18] to estimate crime prevalence rates by mode and gender. Similarly, given a lack of data on safety risk sensitivity, we initialized this parameter by estimating a uniform distribution of decimal values and assigning safety risk sensitivities by drawing from different parts of this distribution for the different SES groups.

The sensitivity analysis was designed to test the sensitivity of each outcome, that is, mode share, travel time and proportion of agents meeting weekly PA recommendations, to a) small changes in the assignment of safety risk sensitivities by SES, and b) variations in the gender and mode-specific crime prevalence rates (i.e., varying the prevalence by 10% above and below the reference values), holding all other parameter values constant (Table 11).

| **TABLE 11 – Sensitivity analysis design** | | |
| --- | --- | --- |
| **Sensitivity scenario** | **Safety risk sensitivity** | **Crime prevalence** |
| Baseline (reference) value | $\left\{ s_{i}\in\{0.1, 0.2, 0.3\} \vert{SES}_{i}=1 or 2 \right\}$ $\left\{ s_{i}\in\{0.4, 0.5, 0.6, 0.7\} \vert{SES}_{i}=3 or 4 \right\}$  $\left\{ s_{i}\in\{0.8, 0.9, 1\} \vert{SES}_{i}=5 or 6 \right\}$ | **Car**: male=0.007% & female=0.008%;  **Motorcycle**: male=0.007% & female=0.003%;  **BRT**: male=0.027% & female=0.048%;  **Bus**: male=0.011% & female=0.017%;  **Bike**: male=0.370% & female=0.130%;  **Walking**: male=0.328% & female=0.590% |
| Wider safety risk distribution | $\left\{ s_{i}\in\{0.1, 0.2, 0.3, 0.4\} \vert{SES}_{i}=1 or 2 \right\}$ $\left\{ s_{i}\in\{0.5, 0.6\} \vert{SES}_{i}=3 or 4 \right\}$  $\left\{ s_{i}\in\{0.7, 0.8, 0.9, 1\} \vert{SES}_{i}=5 or 6 \right\}$ | Baseline values |
| Narrower safety risk distribution | $\left\{ s_{i}\in\{0.1, 0.2\} \vert{SES}_{i}=1 or 2 \right\}$ $\left\{ s_{i}\in\{0.3, 0.4, 0.5, 0.6, 0.7, 0.8\} \vert{SES}_{i}=3 or 4 \right\}$  $\left\{ s_{i}\in\{0.9, 1\} \vert{SES}_{i}=5 or 6 \right\}$ | Baseline values |
| Crime prevalence  10% below baseline | Baseline values | Baseline values – 10% |
| Crime prevalence  10% above baseline | Baseline values | Baseline values + 10% |

The findings of the sensitivity analysis suggest that model outcomes, including mode share, travel time and proportion of the population meeting weekly physical activity guidelines (Figures 8-10), are robust and insensitive to uncertainty in the crime prevalence estimates, and the specification and assignment of safety risk sensitivities by SES. That is, the sensitivity scenarios look almost identical to the baseline scenario shown in the black box of Figures 8-10.


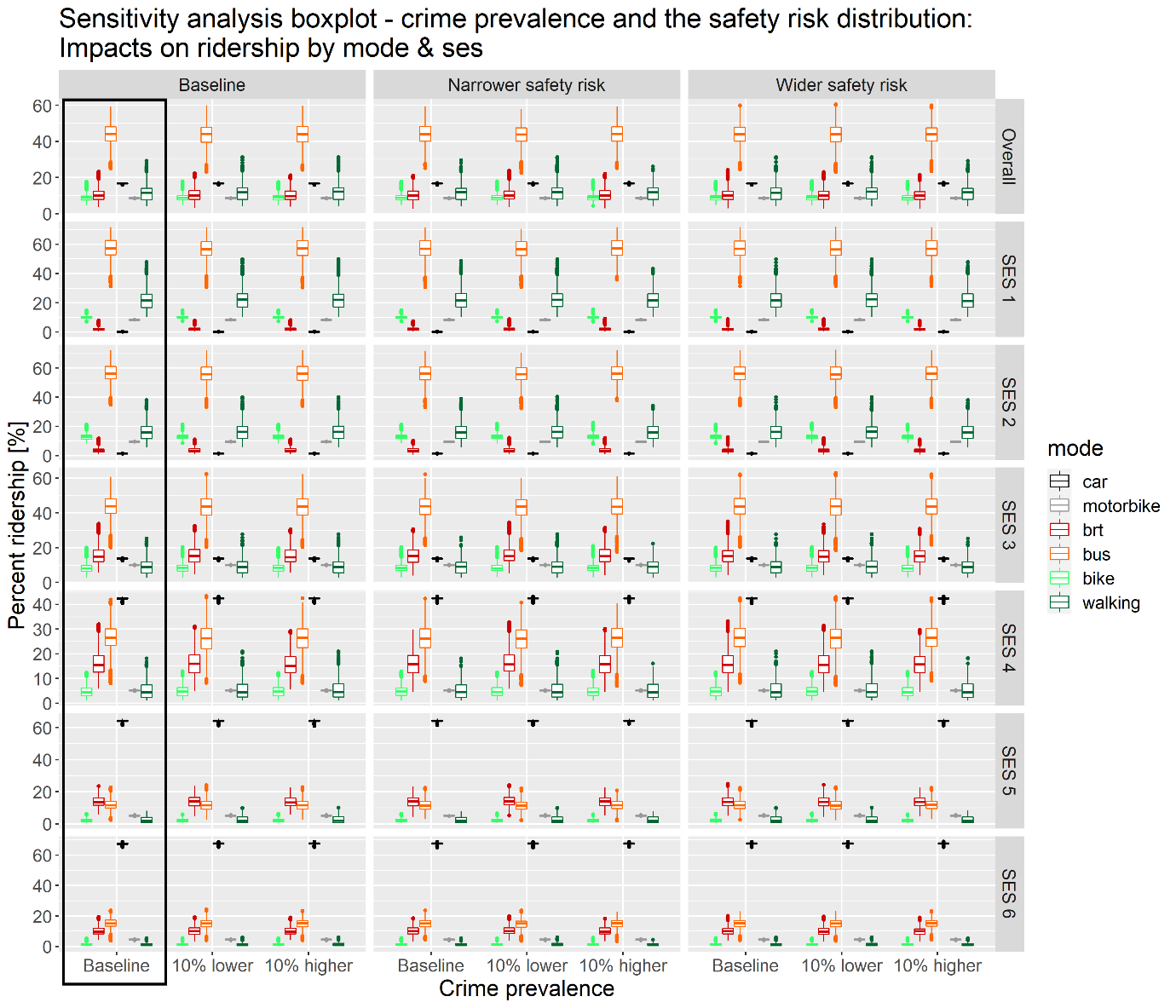


**Figure 8:** Percent ridership (y-axis), by mode (colors), for different crime prevalence sensitivity scenarios (x-axis), including baseline, 10% lower and 10% higher than baseline crime prevalence, and different safety risk sensitivity distribution scenarios (i.e., baseline, narrower, and wider) shown in x-axis facets, overall and by SES (y-axis facets). The left most column/ black box, represents the baseline ridership of each mode, by SES.


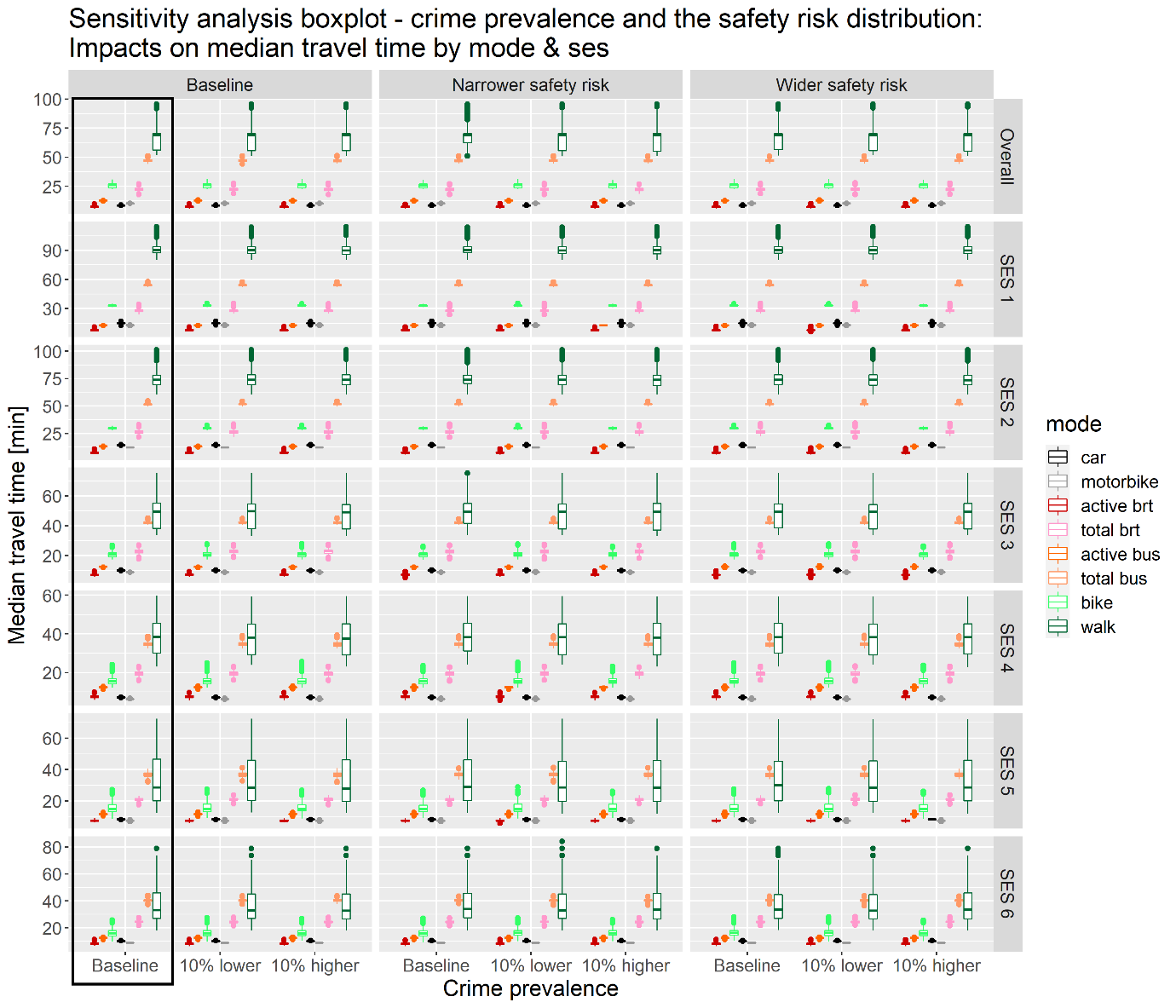


**Figure 9:** Median travel time (y-axis), by mode (colors), for different crime prevalence sensitivity scenarios (x-axis), including baseline, 10% lower and 10% higher than baseline crime prevalence, and different safety risk sensitivity distribution scenarios (i.e., baseline, narrower, and wider) shown in x-axis facets, overall and by SES (y-axis facets). The left most column/ black box, represents the baseline median travel time for each mode, by SES.


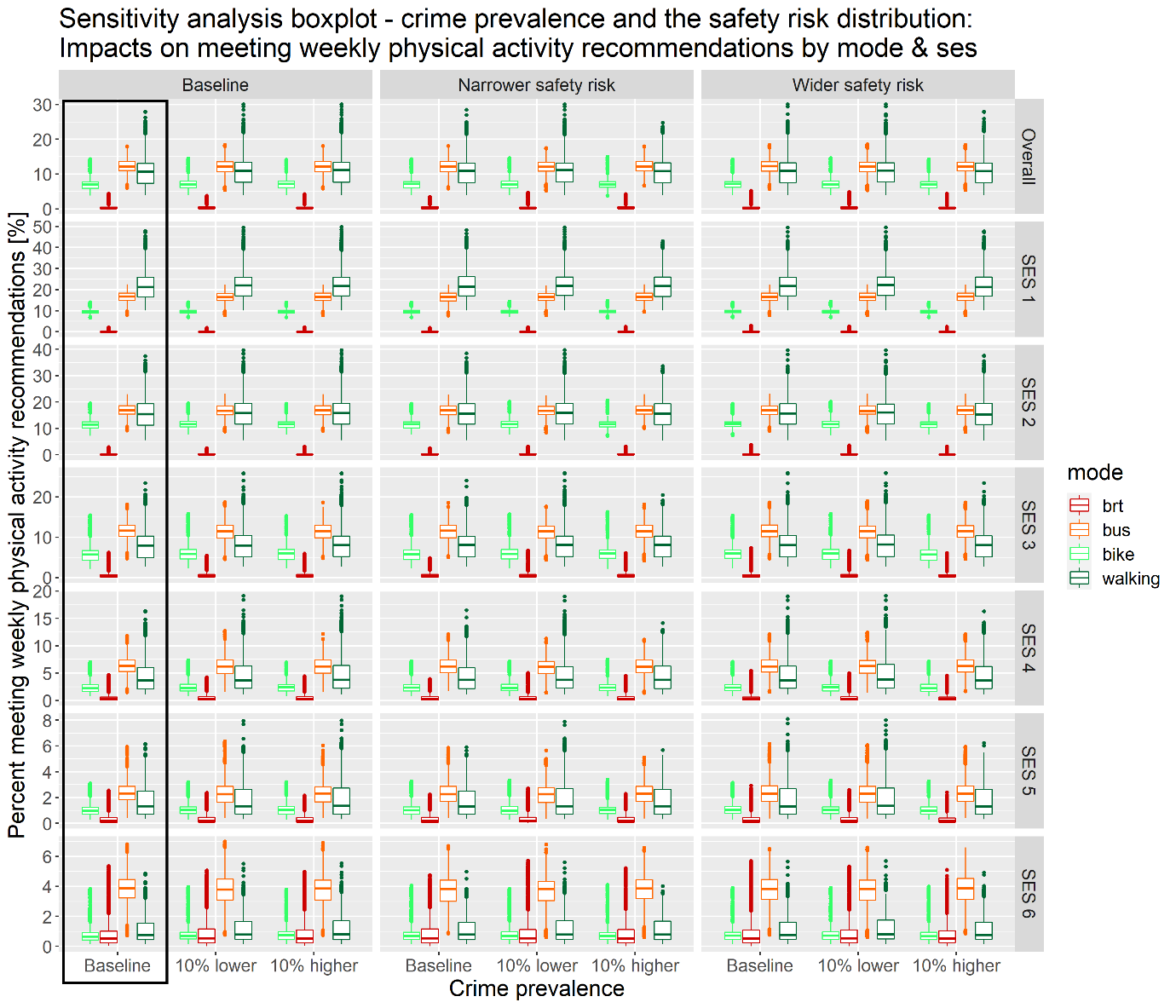


**Figure 10:** Percent meeting weekly physical activity recommendations (y-axis), by mode (colors), for different crime prevalence sensitivity scenarios (x-axis), including baseline, 10% lower and 10% higher than baseline crime prevalence, and different safety risk sensitivity distribution scenarios (i.e., baseline, narrower, and wider) shown in x-axis facets, overall and by SES (y-axis facets). The left most column/ black box, represents the baseline prevalence of meeting weekly physical activity guidelines by mode and SES.

## References

1. Secretaria Distrital de Movilidad Bogotá. *Household Travel Survey 2019*. 2019 [cited 2020 11 June]; Available from: <https://www.simur.gov.co/portal-simur/datos-del-sector/encuestas-de-movilidad/>.

2. Parker, R.N. and R. Fenwick, *The Pareto Curve and Its Utility for Open-Ended Income Distributions in Survey Research.* Social Forces, 1983. **61**(3): p. 872-885.

3. Delignette-Muller, M.L. and C. Dutang, *fitdistrplus: An R package for fitting distributions.* Journal of Statistical Software, 2015. **64**(4): p. 1 - 34.

4. R Core Team, *A language and environment for statistical computing*. 2013, R Foundation for Statistical Computing, Vienna, Austria.

5. QGIS Development Team, *QGIS Geographic Information System*. 2021, Open Source Geospatial Foundation Project.

6. Secretaría Distrital de Planeación de Bogotá. *Bogotá D.C. Stratification Block*. 2019 [cited 2020 17 December 2020]; Available from: <https://datosabiertos.bogota.gov.co/dataset/manzana-estratificacion-bogota-d-c>.

7. Guzman, L.A. and D. Oviedo, *Accessibility, affordability and equity: Assessing ‘pro-poor’ public transport subsidies in Bogotá.* Transport Policy, 2018. **68**: p. 37-51.

8. Oviedo Dávila, N., *Routes to formality: transport accessibility, skills, and labour markets in Bogotá*. 2017, The London School of Economics and Political Science: Latin America and Caribbean.

9. Oviedo, D. and L. Guzman, *Transportation Planning and Development in Bogotá*, in *The Routledge Handbook of Planning Megacities in the Global South*. 2020, Routledge. p. 14.

10. Cantillo-Garcia, V., L.A. Guzman, and J. Arellana, *Socioeconomic strata as proxy variable for household income in transportation research. Evaluation for Bogotá, Medellín, Cali and Barranquilla.* DYNA, 2019. **86**(211): p. 258-267.

11. Meeder, M., T. Aebi, and U. Weidmann, *The influence of slope on walking activity and the pedestrian modal share.* Transportation Research Procedia, 2017. **27**: p. 141-147.

12. Rodrı́guez, D.A. and J. Joo, *The relationship between non-motorized mode choice and the local physical environment.* Transportation Research Part D: Transport and Environment, 2004. **9**(2): p. 151-173.

13. Policia Nacional De Colombia. *Sexual crimes 2015*. 2015a; Available from: <https://www.policia.gov.co/contenido/delitos-sexuales-2015>.

14. Policia Nacional De Colombia. *Homicides 2015*. 2015b; Available from: <https://www.policia.gov.co/contenido/homicidos-2015>.

15. Policia Nacional De Colombia. *Personal theft 2015*. 2015c; Available from: <https://www.policia.gov.co/contenido/hurto-personas-2015>.

16. Policia Nacional De Colombia. *Personal injuries*. 2015d; Available from: <https://www.policia.gov.co/contenido/lesiones-personales-2015>.

17. Policia Nacional De Colombia. *Gender-based violence*. 2015e; Available from: <https://www.policia.gov.co/contenido/violencia-intrafamiliar-2015>.

18. Prudencia Bogota. *Mobility survey 2015*. 2015; Available from: <https://www.simur.gov.co/portal-simur/datos-del-sector/encuestas-de-movilidad/>.

19. Secretaria Distrital de Movilidad Bogotá. *SITP stops Bogotá D.C*. 2019 [cited 2020 5 June]; Available from: <https://datos.movilidadbogota.gov.co/datasets/paraderos-sitp-bogot%C3%A1-d-c/explore?location=4.613945%2C-74.115079%2C11.54>.

20. Transmilenio S.A. *Estaciones troncales de Transmileno*. 2019 [cited 2020 5 June]; Available from: <https://datosabiertos-transmilenio.hub.arcgis.com/datasets/estaciones-troncales-de-transmilenio/explore?location=4.650200%2C-74.125000%2C12.33>.
